# Otitis media sequelae and hearing in adolescence after administration of an 11-valent conjugate pneumococcal vaccine in infancy: a prospective cohort study with long-term follow-up of the ARIVAC trial

**DOI:** 10.1016/S2352-4642(24)00128-7

**Published:** 2024-09

**Authors:** Eric A F Simões, Phyllis Carosone-Link, Diozele M Sanvictores, Kristin M Uhler, Marilla Lucero, Veronica Tallo, Kenny H Chan

**Affiliations:** a Department of Epidemiology, Center for Global Health, Colorado School of Public Health, Aurora, CO, USA; b Department of Pediatrics, Children's Hospital Colorado, Aurora, CO, USA; c Department of Pediatric Otorhinolaryngology, Children's Hospital Colorado, Aurora, CO, USA; d Department of Audiology, Speech-Pathology, and Learning, Children's Hospital Colorado, Aurora, CO, USA; e Department of Pediatrics, University of Colorado School of Medicine, Aurora, CO, USA; f Department of Otorhinolaryngology-Head and Neck Surgery, University of Colorado School of Medicine, Aurora, CO, USA; g Department of Physical Medicine and Rehabilitation, University of Colorado School of Medicine, Aurora, CO, USA; h Department of Clinical Trials, Epidemiology, and Biostatistics, Research Institute for Tropical Medicine, Manila, Philippines

## Abstract

**Background:**

Pneumococcal conjugate vaccines (PCVs) have been shown in randomised controlled trials and epidemiological studies to prevent acute otitis media caused by vaccine serotype pneumococci, although their role in preventing complications of acute otitis media is less clear. We hypothesised that the 11-valent PCV would reduce the long-term sequelae of acute otitis media, including moderate-to-severe ear disease and hearing loss.

**Methods:**

This prospective cohort study, referred to as 11PCV study, included follow-up after 16–20 years of children previously enrolled in 2000–04, at age 6 weeks to 6 months, in the randomised, placebo-controlled, ARIVAC trial of 11-valent PCV for the prevention of radiographical pneumonia. The ARIVAC trial and this 11PCV study were conducted at six study centres in Bohol, Philippines. Ear disease was classified using video-otoscopy review and observations derived from the ear exam. The final classification of the worst ear disease was mild (ie, acute otitis media, otitis media with effusion, healed perforation, or tympanosclerosis), moderate (ie, dry perforation or adhesive otitis media), or severe (chronic suppurative otitis media). Hearing loss was assessed following a standard schema and classified according to the worst ear as mild (>15 to 30 dB puretone average) or moderate-to-profound (>30 dB pure tone average). We calculated the relative and absolute risk reduction in the primary outcome of moderate-to-severe ear disease and the secondary outcomes of mild or moderate-to-profound hearing loss in adolescents who previously received the 11-valent PCV compared with those who received placebo during infancy in ARIVAC.

**Findings:**

Of the 15 593 children assessed for eligibility in ARIVAC, 12 194 were randomly assigned and 8926 were alive and could be located for enrolment in this 11PCV study between Sept 19, 2016, and Dec 13, 2019. 8321 (4188 in the vaccine group and 4133 in the placebo group) completed follow-up of the 11PCV study by March 30, 2020, and had sufficient data to classify ear disease and be included in the primary outcome analysis. The primary outcome of the absolute risk reduction in moderate-to-severe ear disease in the vaccine group (310 [7·4%] of 4188) versus those in the placebo group (356 [8·6%] of 4133) was 1·2% (95% CI 0·0–2·4; p=0·046) and the relative risk reduction was 14·1% (0·0 to 26·0). There were no differences in secondary outcomes of mild hearing loss or moderate-to-profound hearing loss between the vaccine and placebo groups.

**Interpretation:**

The absolute risk reduction for moderate-to-severe ear disease in adolescence of 1·2% (12 per 1000 children) was almost three times higher than the 0·45% reduction (4·5 per 1000 children) in radiographical pneumonia in the first 2 years of life shown in ARIVAC. Administration of 11-valent PCV in infancy was associated with absolute and relative risk reductions in the sequelae of acute otitis media 16–20 years after the original ARIVAC trial.

**Funding:**

Bill & Melinda Gates Foundation.

## Introduction

According to WHO, 430 million people worldwide have moderate-to-profound hearing loss in the better hearing ear, of whom two-thirds live in Asia and Africa.^1^ Deafness and hearing impairment are major forms of disability in low-income and middle-income countries (LMICs) that cause enormous social, educational, and vocational problems.^1^ Chronic middle ear infection is the main cause of mild-to-moderate hearing impairment in children. Middle ear diseases are a common cause of preventable hearing loss, and chronic suppurative otitis media is the most common cause in LMICs.^2^ Chronic suppurative otitis media, otitis media with effusion, adhesive otitis media, scarring of tympanic membranes, and hearing loss are sequelae of acute otitis media. Long-term middle ear effects include tympanic membrane perforation, ossicular erosion, and cholesteatoma, and inner ear effects include sensory–neural hearing loss, especially at higher frequencies.^3^ Since chronic suppurative otitis media is most likely to occur after episodes of incompletely or inadequately treated bacterial acute otitis media, the problem of underdiagnosed acute otitis media could have a direct impact on the burden of chronic otitis media and its sequelae.^4^ Before the introduction of pneumococcal vaccines, the pneumococcus was a major bacterial cause of acute otitis media.^5^

**Research in context**

**Evidence before this study**

Although the role of pneumococcal vaccines in reducing pneumococcal invasive disease, pneumonia, and nasopharyngeal colonisation is well known, their role in preventing acute otitis media and its complications is less clear. We searched Medline, Embase, and the Global Health databases for randomised trials published between Jan 1, 1995, and Sept 21, 2023, on the efficacy of pneumococcal vaccines on otitis media sequelae and used a combination of search terms and related words including “pneumococcal vaccine” AND “chronic otitis media” AND “trial”. No constraints on language were applied. We only found one study comparing pneumococcal carriage and ear discharge in Australian Aboriginal Peoples whose children received the 7-valent pneumococcal conjugate vaccine (7PCV) or PHiD-CV10 with a 7-month follow-up, which showed a small but insignificant relative reduction in ear discharge (22·9% [95% CI –12·2 to 47·1]; 47 [10·4%] of 451 in the PHiD-CV10 group *vs* 60 [13·5%] of 444 in the 7PCV group). A 2020 Cochrane review of 11 randomised controlled trials (n=60 733 children) showed a 11·0–53·0% reduction in pneumococcal acute otitis media in those who received the pneumococcal vaccine compared with controls. The effect on all-cause acute otitis media and recurrent otitis media was less clear. Secondary analyses of vaccine trials in the USA and Finland indicated that pneumococcal vaccines led to fewer ventilation tube insertions for chronic recurrent otitis media.

**Added value of this study**

No trials have studied the efficacy of pneumococcal conjugate vaccine in preventing the most serious sequelae of acute otitis media, namely chronic suppurative otitis media and chronically spontaneously perforated tympanic membranes in high-income countries or in low-income and middle-income countries, where most of the burden of these sequelae occurs. Earlier, we conducted a randomised placebo-controlled trial (1:1) of an 11-valent PCV (11PCV) in 12 191 Filipino children between 2000 and 2004, in which we showed a relative reduction of 22·9% (95% CI –1·1 to 41·2) in radiographical pneumonia. Based on findings of earlier pneumococcal vaccine trials in relation to acute otitis media, recurrent acute otitis media, and ventilation tube insertions, we hypothesised that 11PCV would reduce the sequelae of acute otitis media, namely adhesive and chronic suppurative otitis media. Between 2016 and 2020, we followed up children enrolled in the original ARIVAC trial and showed that those with acute otitis media who received the 11PCV had reduced moderate-to-severe otitis media sequelae (ie, chronic suppurative otitis media, adhesive otitis media, or tympanic perforation), with an absolute risk reduction of 1·2% (0·0 to 2·4) and relative risk reduction of 14·1% (0·0 to 26·0), compared with children who received placebo. There was no association of 11PCV on overall hearing outcomes. However, children with moderate-to-severe ear disease and mild hearing loss who received 11PCV had an absolute risk reduction of 0·6% (0·1 to 1·1) and a relative risk reduction of 35·6% (5·0 to 56·4).

**Implications of all the available evidence**

Given the well known consequences of complicated chronic otitis media on hearing and cognitive development, and—later on—school performance, this association between receipt of a pneumococcal vaccine during infancy and reduced debilitating sequelae of chronic otitis media during adolescence might be of importance in the reduction of the overall burden of disease caused by pneumococcus.

Although the role of pneumococcal vaccines in reducing invasive pneumococcal disease, pneumonia, and pneumococcal colonisation of the nasopharynx is known,^6^ their role in preventing acute otitis media and its complications is less clear. A recent Cochrane Review of 11 randomised controlled trials (RCTs; n=60 733 children)^7^ showed that pneumococcal acute otitis media was reduced by pneumococcal vaccines (relative risk reduction of 20·0% [95% CI 7·0 to 31·0] by the CRM197-PCV7 vaccine,^8^ 25·0% [11·0 to 37·0] by the OMPC-PCV7 vaccine,^9^ and 53·0% [16·0 to 74·0] and 52·0% [37·0 to 63·0] for the PHiD CV10 and CV11 vaccines).^10^, ^11^ The effect on all-cause acute otitis media was less clear,^8^, ^9^, ^12^, ^13^, ^14^ as was the effect on recurrent acute otitis media (relative risk reduction 9·0% [–12·0 to 27·0]).^8^, ^14^ Secondary analyses of vaccine trials in the USA (CRM197-PCV7)^14^ and Finland (PHiD-CV10)^15^ indicated that these vaccines led to fewer ventilation tube insertions for chronic, recurrent otitis media. None of the pneumococcal vaccine trials looked at the risk reduction of the most serious sequelae of acute otitis media (ie, chronic suppurative otitis media and chronically spontaneously perforated tympanic membranes) in high-income countries or in LMICs where most of the burden of these sequelae occurs.

We previously conducted the randomised, placebo-controlled ARIVAC trial of an 11-valent pneumococcal conjugate vaccine (11PCV) in 12 191 Filipino infants between 2000 and 2004, in which we showed a relative reduction of 22·9% (95% CI –1·1 to 41·2) in radiographical pneumonia in infants younger than 2 years.^16^ We hypothesised that 11PCV would also reduce the sequelae of acute otitis media. Between 2016 and 2020, we followed up adolescents who were enrolled in the trial during infancy, and hypothesised that the prevalence of otitis media with effusion, chronic suppurative otitis media, and hearing impairment would be reduced in adolescents vaccinated with 11PCV compared with those who were not vaccinated with 11PCV. Preventing otitis media with effusion and chronic suppurative otitis media might result in better hearing and more advantageous speech and language development in children who received 11PCV, leading to improved school performance. The primary objective of the overall adolescent follow-up study was to compare school performance and school dropout rates in children who did and who did not receive 11PCV during infancy.^17^ In this paper we present the secondary objectives of this follow-up study to evaluate the proximate biological pathway by which 11PCV administered in infancy might affect cognitive performance, and might ultimately result in diminished school performance and higher school dropout rates through acute otitis media sequelae and hearing loss. In the analysis presented here we aimed to compare moderate-to-severe ear disease and hearing loss in adolescents who had or had not received 11PCV during infancy.

## Methods

### Study design and participants

This prospective cohort study (herein referred to as the 11PCV study) included follow-up after 16–20 years of children previously enrolled in a randomised, placebo-controlled trial at age 6 weeks to 6 months, conducted in Bohol, Philippines, in 2000–04 (ARIVAC trial; ISRCTN62323832).^16^

Study participants in ARIVAC had their homes geocoded. These coordinates were used to track families and children for this 11PCV follow-up study. All parents provided written informed consent and adolescents provided written assent (those older than 18 years provided written informed consent). The study protocol is available online^18^ and the statistical analysis plan is included in the appendix (pp 1–66). This study was approved by the institutional review boards at the Regional Institute of Tropical Medicine (Manila, Philippines; protocol 2016–20–2) and the Colorado Multiple Institutional Review Board (protocol 16–0473; Aurora, CO, USA).

### Procedures

Parents were asked to complete a detailed social demographic and illness questionnaire, developed specifically for this 11PCV follow-up study, and children received ear exams at six study centres in Bohol, Philippines. The child's sex was recorded in ARIVAC and confirmed during this 11PCV study. Ethnicity was also recorded during the demographic questionnaire. Trained study nurses^19^ examined participants' ears, and tympanic membrane images were recorded using a video otoscope (JedMed, St Louis, MO, USA). Participants underwent tympanometry and distortion product otoacoustic emission (Path Sentiero, Path Medical, Germering, Germany) and audiometric testing using the HearScreen test (mHealth Studio, Pretoria, South Africa). When wax or otorrhoea prevented conducting a valid test, the adolescent was referred to an otolaryngologist in Bohol to clear the ear and provide treatment as deemed clinically necessary, after which the participant returned to complete their ear exam. Those who did not meet the threshold on the distortion product otoacoustic emission or HearScreen test were referred to the Bohol Hearing Center (Bohol, Philippines) for formal audiometric testing in a soundproof room.

The study nurses in Bohol uploaded data into a REDCap database that could be accessed by the US team for the classification of ear disease. One of the coauthors (KHC) trained and supervised all ear, nose, and throat physician assistants in the USA to identify the signs and symptoms from the video-otoscopies of the study participants. Ear disease was classified as previously described^18^ using the video-otoscopy review and observations derived from the ear exam (ie, signs and symptoms from the ear exam visit [eg, ear pain or redness of the tympanic membrane], tympanogram parameters, and any observations and diagnoses made by the otolaryngologist in Bohol). The US investigators who defined the ear disease classification scheme^18^ consisted of the otolaryngologist (KHC), audiologist (KMU), and principal investigator (EAFS). This classification scheme incorporated standard clinical methods and metrics from the literature. If the adolescent's ears could not be classified due to obscured tympanic membrane or no video-otoscopy due to the presence of otorrhoea, an alternative classification scheme was used (appendix p 67) that was developed by the otolaryngologist (KHC) at Children's Hospital Colorado (Aurora, CO, USA). Only children who were referred to the otolaryngologist in Bohol to clear and treat the child's ear had the potential to be diagnosed by this otolaryngologist.

For the primary ear disease outcome, the final classification of the worst ear disease was mild (ie, acute otitis media, otitis media with effusion, healed perforation, or tympanosclerosis), moderate (ie, dry perforation or adhesive otitis media), and severe (chronic suppurative otitis media including otorrhoea, granulation tissue, or cholesteatoma).^18^ In cases of some missing exam data (eg, no tympanogram or tympanic membrane obscured), all the available data were reviewed and a diagnosis made according to the flowchart in the appendix (p 67). The complex schema for the hearing assessment using the HearScreen, distortion product otoacoustic emission, and formal audiometric assessment is shown in the appendix (pp 27–28, 38–40). For the secondary hearing loss outcomes, mild hearing loss was defined as more than 15 and up to 30 decibels (dB) pure tone average in the worst ear, and moderate-to-profound hearing loss as more than 30 dB pure tone average in the worst ear.

In ARIVAC, the investigational 11PCV prepared and manufactured by Sanofi Pasteur (Lyon, France) contained 1 μg of *Streptococcus pneumoniae* capsular polysaccharide conjugated to tetanus toxoid for types 1, 4, 5, 7F, 9V, 19F, and 23F; 3 μg of polysaccharide of types 3, 14, and 18C conjugated to diphtheria toxoid; and 10 μg of polysaccharide of type 6B conjugated to the diphtheria toxoid. Saline was used as the placebo. Block randomisation was used to allocate participants to the vaccine or placebo groups. Participants and investigators in ARIVAC were masked to treatment allocation. The participants' parents were informed of the allocation at the end of ARIVAC^16^ and so parents and participants were no longer masked to treatment allocation for this 11PCV follow-up study. All ear disease classification, hearing classification, data entry, and data cleaning, were done by investigators and data managers while masked to vaccine status, which was only merged with the rest of the study data during the data analysis phase.

### Statistical analysis

Assuming a rate of otitis media with effusion plus chronic suppurative otitis media of 5% in unvaccinated children (based on the rate from our previous study in Bali),^20^ 5000 of the ARIVAC study participants would need to be followed up to detect a 40% relative risk reduction in otitis media with effusion and chronic suppurative otitis media with 80% power and a two-tailed 5% false positive α rate. For 90% power, 7000 of the ARIVAC study participants would need to be followed up in this 11PCV study.

The primary objective for this analysis was to compare moderate-to-severe ear disease during adolescence between the vaccine and placebo groups. The secondary objective was to compare mild hearing loss or moderate-to-profound hearing loss in the worst ear during adolescence between the vaccine and placebo groups. The tertiary post-hoc objectives were the comparison of reductions in mild or moderate-to-profound hearing loss between the vaccine and placebo groups in adolescents with moderate-to-severe ear disease. Relative and absolute risk reductions with 95% CIs^21^ in the vaccine group compared with the placebo group for all outcomes were computed using JavaStat.

Comparisons of categorical demographic characteristics were made for participants enrolled in this 11PCV study and those in ARIVAC who were lost to follow-up and not available for enrolment in this 11PCV study to determine whether the current 11PCV study sample was representative of the original ARIVAC sample. Demographic characteristics and potential risk factors were also compared using univariable statistics between vaccine and placebo groups in the 11PCV study. The socioeconomic index was computed using the Demographic and Health Survey Wealth Index. Diagnostic codes for otitis media (chronic suppurative otitis media, otitis media with effusion, and acute otitis media) were included in the ARIVAC database for children who were admitted to hospital or were seen in a study clinic. The occurrences of otitis media before age 2 years recorded during ARIVAC were compared with occurrences of otitis media reported in this 11PCV follow-up study using odds ratios and 95% CIs. Descriptive statistics of pure tone averages for children with and without hearing loss were compared separately by the type of moderate-to-severe ear disease. Statistical methods used for these comparisons are described in the appendix (p 68). A p value of less than 0·05 was used as the significance threshold.

The presentation of results differs from the original statistical analysis plan in the following ways: (1) since examination of demographic and risk factors did not show any statistically significant difference between the vaccine and placebo groups, multivariable analysis with odds ratios was not performed; instead a simple univariable comparison presenting data as absolute risk reduction and relative risk reduction was presented for the primary analysis; (2) associations between otitis media diagnosed in ARIVAC and ear disease classification in this 11PCV study were examined; (3) a tertiary objective of the relative and absolute risk reductions in children with ear disease and hearing loss who received vaccine compared with placebo was added.

### Role of the funding source

The funder of the study had no role in study design, data collection, data analysis, data interpretation, or writing of the report.

## Results

Of the 15 593 children assessed for eligibility in ARIVAC during 2000–03 and followed up until completion of the trial at age 24 months or until December, 2004 (whichever came first), 12 194 were randomly assigned and 8926 were alive and could be located for enrolment in this 11PCV study between Sept 19, 2016, and Dec 13, 2019 (figure). 8362 children completed follow-up ear exams by March 30, 2020. Of these 8362 participants, 8321 (4188 in the vaccine group and 4133 in the placebo group) had sufficient data to classify ear disease and be included in the primary outcome analysis. 15 793 children's ears were classified as per the definitions in the statistical analysis plan (appendix pp 37–38), 82 ears were non-classifiable, and 849 ears were classified by an alternative method (appendix p 67). 13 children's ears in three nodes had an audiological review to classify the tympanogram (in cases for which the tympanogram type was used primarily to classify ear disease type). 41 ears were classified for ear disease type by an otolaryngologist (KHC) at the Children's Hospital Colorado after review of all available data.

**Figure:**
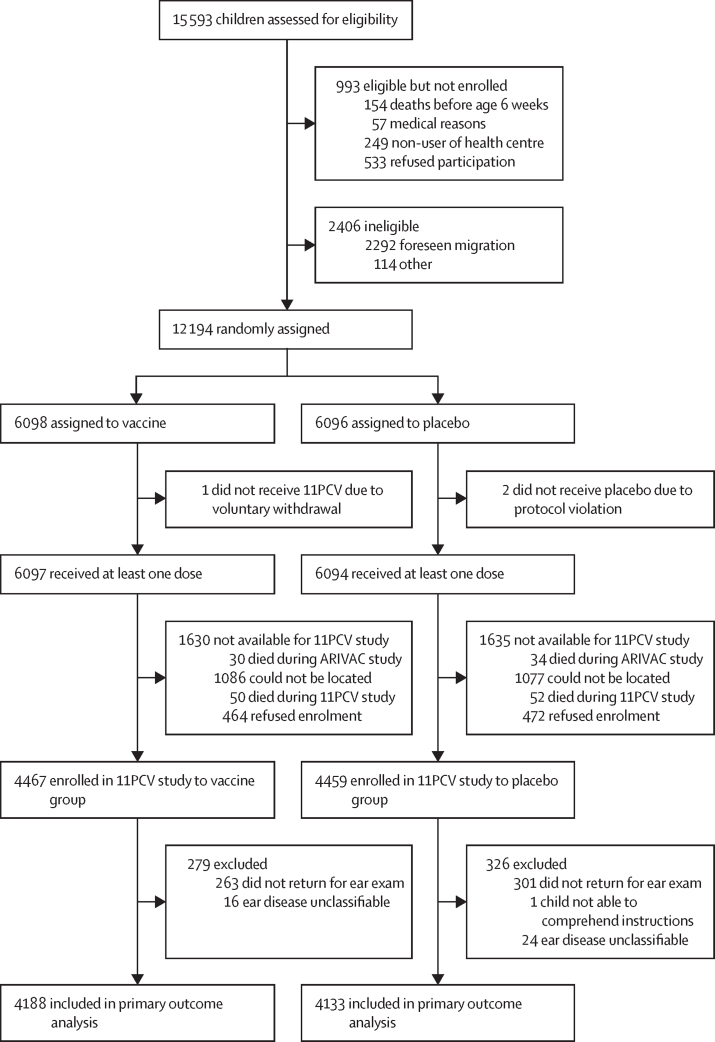
Study profile

Adolescents who could not be located or declined to participate in this 11PCV study and were presumed to be alive (1550 in the vaccine *vs* 1549 in the placebo groups; appendix p 69) did not differ by sex from those who were located and enrolled in 11PCV study follow-up (4467 in the vaccine *vs* 4459 in the placebo groups; figure), but did differ by year of birth, city or town of residence during ARIVAC, and mother's employment status (appendix p 69), as well as mother's education and the number of siblings at enrolment into ARIVAC (appendix p 70).

The demographic characteristics, potential risk factors, and medical history of adolescents were generally well balanced between the vaccine and placebo groups (table 1; appendix p 71). The median age of adolescents at the interview was 16·3 years (SD 1·3). All participants included in this study were Asian.

**Table 1:** Demographic characteristics, potential risk factors, and medical history of children by vaccine status

			**11PCV group (n=4188)**	**Placebo group (n=4133)**	p value*
Demographics and potential risk factors					
	Child resides in urban area		1674 (40·0%)	1694 (41·0%)	0·36
	Male sex†		2130 (50·9%)	2090 (50·6%)	0·81
	Female sex†		2058 (49·1%)	2043 (49·4%)	0·81
	Family has health insurance		2991/4141 (72·2%)	2955/4074 (72·5%)	0·78
	Child smokes cigarettes		74/4186 (1·8%)	62/4132 (1·5%)	0·38
	Family has any of these animals around the house (cat, dog, bird, rabbit, chicken, goat, cow, horse, water buffalo, rat, mouse, or cockroaches)		3985/4184 (95·2%)	3955/4125 (95·9%)	0·18
	Child ever swam		3469/4080 (85·0%)	3407/4013 (84·9%)	0·90
		Child still swims	3233/3467‡ (93·3%)	3163/3405‡ (92·9%)	0·59
	Child has difficulty hearing		141/4169 (3·4%)	141/4120 (3·4%)	0·97
		Hearing problem continuous	92/140‡ (65·7%)	90/138‡ (65·2%)	1·00
		Sought medical care for this condition	26/140‡ (18·6%)	33/141 (23·4%)	0·40
		Child had surgery for this condition	0/141	1/141 (0·7%)	1·00§
Child has allergy symptoms					
	Allergy to animals		234/4153 (5·6%)	201/4091 (4·9%)	0·16
	Itchy nose		72 (1·7%)	56 (1·4%)	0·21
	Watery, red, or itchy eyes		29 (0·7%)	28 (0·7%)	1·00
	Rhinitis		427/4169 (10·2%)	465/4117 (11·3%)	0·13
Either parent has a history of ear drainage			104 (2·5%)	127 (3·1%)	0·12
Child had a disease diagnosed by a medical professional					
	Measles		472/4147 (11·4%)	518/4099 (12·6%)	0·090
	Heart condition		13/4183 (0·3%)	5/4125 (0·1%)	0·10
Parent recognised any ear problems in child in the past (at any point)					
	Ear discharge		204/849‡ (24·0%)	227/875 (25·9%)	0·39
		Continuous discharge	37/204 (18·1%)	51/227 (22·5%)	0·32
		Sought medical care for this condition	116/203‡ (57·1%)	140/227 (61·7%)	0·39
	Perforation of the eardrum		30/849‡ (3·5%)	38/875 (4·3%)	0·46
	Hearing loss		127/850 (14·9%)	120/875 (13·7%)	0·51
Child's age at interview, years			16·5 (1·3); 16 (15–17)	16·0 (1·3); 16 (15–17)	0·77¶
Child-years of study follow-up			16·4 (1·3); 16 (15–17)	16·0 (1·3); 16 (15–17)	0·77¶
Family DHS Wealth Index‖			0·5 (0·7); 0 (0–1)	0·5 (0·7); 0 (0–1)	0·56¶
Number of people in home divided by the number of bedrooms (ie, crowding)**			2·8 (1·7); 2 (2–4)	2·8 (1·7); 2 (2–4)	0·67¶
Number of adults (aged >15 years) living in the house††			3·9 (1·9); 4 (3–5)	3·8 (1·9); 3 (3–5)	0·063¶
Number of household members regularly smoking in the house‡‡			0·5 (0·7); 0 (0–1)	0·5 (0·7); 0 (0–1)	NA
Number of cigarettes smoked by the child per day§§			0·02 (0·1); 0 (0–0)	0·02 (0·1); 0 (0–0)	NA
Number of households with smokers‡‡			0·4 (0·5); 0 (0–1)	0·4 (0·5); 0 (0–1)	0·39¶

Data are n (%), n/N (%), or mean (SD); median (IQR). DHS=Demographic and Health Survey. All children included in this study were Asian. NA=not applicable. 11PCV=11-valent pneumococcal conjugate vaccine.

* Continuity-adjusted p value, except where otherwise indicated.

† Male and female sex was assigned at birth and given by the parents of the infants during enrolment into the ARIVAC study. Sex was verified during enrolment into this 11PCV study.

‡ Missing responses.

§ Fisher's exact test p value.

¶ Student's *t* test results; with large sample sizes the central limit theorem ensures that means will be normally distributed (except for the most skewed distributions, in which case the test will be liberal).

‖ More information about the Wealth Index Construction and steps to constructing the new DHS Wealth Index can be found online.

** n=4180 in the 11PCV group and n=4117 in the placebo group.

†† n=4188 in the 11PCV group and n=4132 in the placebo group.

‡‡ n=4186 in the 11PCV group and n=4128 in the placebo group.

§§ n=4186 in the 11PCV group and n=4132 in the placebo group.

The primary outcome of the absolute risk reduction in moderate-to-severe ear disease in the vaccine group (310 [7·4%] of 4188) compared with the placebo group (356 [8·6%] of 4133) was 1·2% (95% CI 0·0 to 2·4; p=0·046) and the relative risk reduction was 14·1% (0·0 to 26·0; table 2). There were no differences in secondary outcomes of mild or moderate-to-profound hearing loss between the 11PCV and placebo groups. In a subgroup tertiary post-hoc analysis of adolescents with moderate-to-severe ear disease, there was an absolute risk reduction of 0·6% (0·1 to 1·1; p=0·025) for those with mild hearing loss between the vaccine and placebo groups, but this finding was not seen in children with moderate-to-profound hearing loss (–0·1% [–0·5 to 0·3]; p=0·65; table 2). Distributions of children by ear disease severity, components of moderate and severe ear disease, and hearing loss severity by vaccine status are shown in the appendix (pp 72–74).

**Table 2:** Risk reduction by ear disease and hearing loss severity in those who received 11PCV compared with placebo

	**11PCV group**	**Placebo group**	p value*	**Absolute risk reduction (95% CIs)**	**Relative risk reduction (95% CIs)**
**Primary outcome**					
Moderate-to-severe ear disease	310/4188 (7·4%)	356/4133 (8·6%)	0·046	1·2% (0·0 to 2·4)	14·1% (0·0 to 26·0)
**Secondary outcomes**†					
Mild hearing loss	225/4027 (5·6%)	246/3992 (6·2%)	0·29	0·6% (−0·5 to 1·6)	9·3% (−8·5 to 24·2)
Any moderate-to-profound hearing loss	104/4131 (2·5%)	90/4082 (2·2%)	0·39	−0·3% (−1·0 to 0·4)	−14·2% (−52·4 to 14·4)
**Tertiary post-hoc outcomes**†					
Moderate-to-severe ear disease with mild hearing loss	45/4131 (1·1%)	69/4079 (1·7%)	0·025	0·6% (0·1 to 1·1)	35·6% (5·0 to 56·4)
Moderate-to-severe ear disease with moderate-to-profound hearing loss	34/4131 (0·8%)	29/4079 (0·7%)	0·65	−0·1% (−0·5 to 0·3)	−15·8% (−95·0 to 31·2)

Ear disease and hearing loss classifications were taken from the worst ear. 11PCV=11-valent pneumococcal conjugate vaccine.

* Yates' corrected χ^2^ test.

† The denominators are different for secondary and tertiary outcomes, because not all of the children who were classified for ear disease were successfully tested for hearing loss.

Of the 8321 children who were classified for ear disease, 8210 had a successful hearing test and three additional children with unclassifiable ear disease had a successful hearing test. Although parent-reported ear problems in the past (ie, during the ARIVAC study and until the time of the 11PCV follow-up study) did not differ by vaccine group (table 1), diagnosis of otitis media during ARIVAC for children who were admitted to hospital or seen in a study clinic, and who had a diagnostic code, was significantly associated with the ear disease classification in this 11PCV study (table 3). The occurrence during ARIVAC of acute otitis media, otitis media with effusion, or chronic suppurative otitis media was associated with categories of moderate-to-severe ear disease measured in the 11PCV study with follow-up after 16–20 years with ORs of 3·1 (95% CI 1·3–7·2; p=0·0068) for chronic suppurative otitis media or dry or healed perforation, 7·2 (2·8–18·3; p<0·0001) for chronic suppurative otitis media, and 3·4 (1·6–7·3; p=0·0045) for adhesive otitis media. Table 4 shows the reductions in hearing ability in each of the components of moderate-to-severe ear disease compared with those with no ear disease, with the largest hearing loss in children with chronic suppurative otitis media. Of 663 children with hearing impairment, 255 (38·4%) had no evidence of ear disease.

**Table 3:** Association between otitis media diagnoses during the ARIVAC study and type of ear disease classified during this 11PCV follow-up study

	Child had diagnosis of otitis media*during ARIVAC study	**Child did not have a diagnosis of otitis media during ARIVAC study**	**Odds ratio (95% CIs)**	**p value**†
Chronic suppurative otitis media or dry or healed perforation	6/54 (11·1%)	323/8267 (3·9%)	3·1 (1·3–7·2)	0·0068
Chronic suppurative otitis media	5/54 (9·3%)	116/8267 (1·4%)	7·2 (2·8–18·3)	<0·0001
Adhesive otitis media	8/54 (14·8%)	401/8267 (4·9%)	3·4 (1·6–7·3)	0·0045

Each child was classified with the designated type of ear disease during this 11PCV follow-up study. 11PCV=11-valent pneumococcal conjugate vaccine.

* Otitis media diagnoses for ARIVAC study participants who were later enrolled in this 11PCV study.

† Yates' corrected χ^2^ test for association.

**Table 4:** Comparison of hearing loss for the worst ear stratified by ear disease status

	Number of children (n=5240)*	**Pure tone average, mean (SD)**	**Pure tone average, median (IQR)**	**p value**†
No ear disease	4610	11·2 (7·1)	10 (10–10)	..
Dry perforation	135	16·6 (12·2)	10 (10–20)	<0·0001
Adhesive otitis media	404	13·2 (8·2)	10 (10–10)	<0·0001
Active chronic suppurative otitis media	91	25·7 (16·8)	23 (10–35)	<0·0001

* In total 8210 participants had an ear disease classification and a successful hearing test, but 2970 with mild ear disease are not included here (18 with acute otitis media, 96 with otitis media with effusion, 70 with healed perforation, and 2786 with myringosclerosis).

† p value for Wilcoxon rank-sum test *t* approximation; the comparisons were done separately for each type of ear disease classification versus no ear disease.

## Discussion

In this follow-up cohort study of the ARIVAC trial, we found that receiving the 11PCV in infancy was associated with a reduction in moderate-to-severe ear disease in adolescence. Chronic suppurative otitis media was more common than expected based on our previous studies in Indonesia.^20^ The overall rate of sequelae of acute otitis media of 100 per 1000 adolescents in Philippines is almost four times the rate of that in Indonesia, which is reaching the rates seen in other high-risk populations, such as the Australian Aboriginal Peoples^22^ and Native Americans.^23^ The importance of this reduction in moderate-to-severe sequelae of acute otitis media (absolute risk reduction of 12 per 1000 adolescents) and the consequent reduction in mild hearing loss in those with moderate-to-severe ear disease (six per 1000 adolescents) associated with 11PCV vaccination in infancy comes into perspective when compared with the absolute reduction in radiographical pneumonia associated with 11PCV vaccination in the first 2 years of life in the ARIVAC trial (93 of 6013 vaccinated *vs* 120 of 6018 placebo equals 4·5 per 1000 children),^16^ an almost three times higher absolute risk reduction in the burden of ear disease compared with pneumonia. Although the benefits of a pneumococcal vaccine in reducing invasive pneumococcal disease, pneumonia, and mortality have been well documented and quantified in the past 20 years, to our knowledge, this 11PCV study is the first to quantify the association between chronic otitis media during adolescence and pneumococcal vaccine during infancy in the context of long-term follow-up of a PCV vaccine RCT. In ARIVAC,^16^ 11PCV did not lead to a statistically significant reduction in invasive pneumococcal disease or mortality due to the widespread early use of antibiotics in the Philippines. The relative reduction in chronic suppurative otitis media observed in this 11PCV study might be considered to be low but could be associated with a larger reduction in absolute numbers than the reduction in pneumonia associated with 11PCV. Given the lifelong disability of hearing impairment caused by chronic suppurative otitis media and its effects on cognition and school performance,^24^ the reduction in chronic suppurative otitis media associated with 11PCV might be considered a substantial reduction in the overall burden of disease caused by pneumococcus.

We did not find an association of 11PCV with hearing outcomes, despite there being a strong correlation between the chronic sequelae of acute otitis media and hearing impairment. More than a third (38·4%) of children with hearing impairment in our study had no evidence of ear disease. When we excluded these children, we found that 11PCV was associated with a reduction in mild hearing loss in children with moderate-to-severe ear disease. We speculate that the use of headsets and ambient noise pollution in Tagbilaran, the largest town, contributed to this burden of disease and might have overwhelmed the impact of the vaccine on hearing.

Eleven RCTs of pneumococcal vaccines have not unequivocally shown a reduction in all-cause acute otitis media, despite showing statistically significant reductions in pneumococcal acute otitis media,^11^ and only one study from Australia comparing PHiD-CV10 and 13PCV looked at the outcomes in children aged 7 months of acute otitis media, otitis media with effusion, and chronic suppurative otitis media.^25^ Clearly a substantial proportion of acute otitis media is due to viral upper respiratory infections spreading to the middle ear via the Eustachian tube,^24^ which would explain the low overall reduction in acute otitis media in these PCV vaccine trials.^11^ However, numerous studies from countries implementing infant vaccination programmes that include PCVs have shown an indirect effect on reducing acute otitis media,^26^, ^27^ recurrent otitis media,^27^ and tympanostomy tube placement.^26^ This indirect effect has typically been ascribed to the reduction of vaccine-type pneumococcal nasopharyngeal carriage in children and subsequently in the general population.^7^ Even after the ARIVAC trial in Bohol (Philippines) during 2000–04 in which children received the 11PCV, pneumococcal vaccines were not introduced in the Philippines until 2015 when around 30% of the eligible paediatric population received the 13PCV.^28^ This vaccination rate in Philippines has varied between 50% and 73% in the past 5 years.^28^ The follow-up of adolescents in this study started in 2016, so the association of 11PCV with sequelae of acute otitis media shown here was not due to a reduction in circulating pneumococcus.

In ARIVAC, presumably since we administered the vaccine at age 6, 10, and 14 weeks,^16^ vaccine serotype nasopharyngeal carriage would have been reduced in the vaccinees. The fact that early age of onset of first otitis media is a risk factor for recurrent otitis media (an indicator for development of complex otitis media and chronic suppurative otitis media) is well known. Even with few otitis media cases recorded in ARIVAC, there were important associations between otitis media in the first 2 years of life and sequelae of otitis media in adolescents who were followed up after 16 years. Together, these observations provide the most plausible pathway to explain the effect of PCVs administered early in infancy on sequelae in adolescence. The pneumococcal vaccine was associated with reduced acute and recurrent otitis media in the 11PCV study participants, presumably by reducing nasopharyngeal carriage of the more pathogenic vaccine-specific pneumococcal serotypes. Preventing acute perforation, chronic otitis media with effusion, and complex otitis media with effusion in infancy,^29^ which appear to be more dependent on otologically-virulent pneumococcal species than non-typeable *Haemophilus influenzae*,^30^ could result in reduced long-term sequelae shown in this study.

The main limitation here is the long interval between the ARIVAC study and this 11PCV study with inability to follow-up all participants. There were some differences by year of birth, city or town of residence during ARIVAC, and mother's employment status between participants who were located and available for enrolment into the 11PCV follow-up study and those who were not. In addition, there was a 12–15-year delay since ARIVAC was completed and this follow-up, without continuous follow-up of the participants. However, we followed up 8926 (73%) of 12 191 participants from ARIVAC and included 8321 (68%) in this analysis who had complete data. We did find small relative and absolute reductions in the degree and severity of ear disease, but not in overall hearing. Other factors, such as use of ear buds or the effects of ubiquitous noise pollution in the town of Tagbilaran (in which 40·5% of the participants lived), might have influenced hearing outcomes, and we did not examine this limitation further. However, in a post-hoc tertiary analysis, 11PCV was associated with a reduction in mild hearing loss in participants with moderate-to-severe ear disease. The long time between ARIVAC and this follow-up and the loss to follow-up did not impact the study results, which can also be seen in the statistically significant associations between early otitis media shown in the first 2 years of life and the subsequent chronic ear disease shown 12–16 years later.

To conclude, administration of 11PCV in infancy was associated with absolute and relative risk reductions in the sequelae of acute otitis media 16–20 years after the original ARIVAC trial. This reduction was around three times higher than the absolute reduction of the vaccine on radiographical pneumonia in infancy seen in the ARIVAC study. We did not show an association of 11PCV with hearing loss, although in a post-hoc analysis of a subgroup of children with moderate-to-severe ear disease we showed that mild hearing loss was significantly reduced.

Given the huge burden of disease uncovered by the follow-up of participants (8·0% of adolescents had long-term sequelae of acute otitis media) and that this burden (14·1% relative risk reduction in moderate-to-severe ear disease) could be prevented by the pneumococcal vaccine, this study underscores the importance of vaccinating children. Given the low rates of pneumococcal vaccine uptake in several LMICs,^31^ the reduction of a disease with lifelong impact has a substantial public health bearing. The under-recognised burden of chronic otitis media underscores the need for large-scale studies to investigate the potential decrease by pneumococcal vaccination.

For the **Wealth Index Construction** see https://dhsprogram.com/topics/wealth-index/Wealth-Index-Construction.cfm

For more on **constructing the DHS Wealth Index** see https://www.dhsprogram.com/programming/wealth%20index/Steps_to_constructing_the_new_DHS_Wealth_Index.pdf

### Contributors

### Data sharing

Aggregate data that underlie the results reported in this study, the study protocol, and the statistical analysis plan will be available upon publication. Individual participant data, a data dictionary defining each field in the set, and the software code will not be made available. Data will be shared and available from the Corresponding author (eric.simoes@cuanschutz.edu) between 9 months and 36 months after publication to researchers who provide a methodologically sound proposal and whose proposed use of the data—for a meta-analysis—has been approved by an independent review committee and ethical review, and has received clearance by the Regional Institute of Tropical Medicine (Manila, Philippines) and Asian Foundation for Tropical Medicine (Manila, Philippines). Investigator support will be required after approval of a proposal with a signed data access agreement before data will be shared.

## Declaration of interests

EAFS, PC-L, DMS, ML, VT, KMU, and KHC have received grants from the Bill & Melinda Gates Foundation for this study. EAFS reports grants and consulting fees to the institution from Merck and Pfizer; grants to the institution from AstraZeneca, Roche Pharmaceuticals, and Johnson and Johnson; and consulting fees to the institution from Sanofi Pasteur, Cidara Therapeutics, Adiago Therapeutics, and Nuance Pharmaceuticals; manuscript writing support from Pfizer and AstraZeneca; financial support for attending a meeting from AstraZeneca and Pfizer; and participation on a data monitoring committee for Abbvie, GlaxoSmithKline, and the Bill & Melinda Gates Foundation.
