# Supplementary material for: Otitis media sequelae and hearing in adolescence after administration of an 11-valent conjugate pneumococcal vaccine in infancy: a prospective cohort study with long-term follow-up of the ARIVAC trial
Source: Lancet Child Adolesc Health. 2024 Sep;8(9):647–55. doi: 10.1016/S2352-4642(24)00128-7 (PMC11319240; doi:10.1016/S2352-4642(24)00128-7)
Supplement: Supplementary appendix [file mmc1.pdf]

THE LANCET
Child & Adolescent Health

Supplementary appendix

This appendix formed part of the original submission and has been peer reviewed. We post it as supplied by the authors.

Supplement to: Simões EAF, Carosone-Link P, Sanvictores DM, et al. Otitis media sequelae and hearing in adolescence after administration of an 11-valent conjugate pneumococcal vaccine in infancy: a prospective cohort study with long-term follow-up of the ARIVAC trial. *Lancet Child Adolesc Health* 2024; published online July 31. https://doi.org/10.1016/S2352-4642(24)00128-7.

Appendix Materials

Table of Contents

Statistical Analysis Plan……………………………………………………………..…….1

Appendix Figure 1…………………………………………………………………..….67

Appendix Methods..…………………………………………………..……………..…..68

Tables……………………………………….…………………………………69

Appendix Table 1………………………………………………….………….....69

Appendix Table 2………………………………………………………..…..…..70

Appendix Table 3……………………………………………………….....….71

Appendix Table 4……………………………………………...……….….72

Appendix Table 5……………………………………………...……….….73

Appendix Table 6……………………………………………..……………..74

STATISTICAL ANALYSIS PLAN

Impact of an 11 Valent Pneumococcal Vaccine on School Performance, Hearing and Cognitive Development

University of Colorado School of Medicine, Aurora, CO, USA

Research Institute for Tropical Medicine, Manila, Philippines

The Ohio State University, Columbus, OH, USA

STUDY FUNDER

Bill and Melinda Gates Foundation

INVESTIGATORS

Eric A. F. Simões, Veronica Tallo, Marilla Lucero, Joanne De Jesus, Elisabeth Dowling Root, Kenny Chan, Andrea S. Miele, Kristin Uhler, Phyllis Carosone-Link, Diozele Sanvictores

PREPARED BY

PCV 11 Follow-up Study Team

Biostatistician: Elisabeth Root

STUDY INITIATION DATE:

1st September 2016

FOLLOW-UP END DATE:

30th September 2019

Table of Contents

Study objectives 6

1.1 Primary objectives.......... 6

1.1.1 To measure differences in academic performance between 11 PCV and placebo groups. 6

1.1.2 To measure differences in permanent school dropout rates between 11 PCV and placebo groups. 6

1.2 Secondary objectives 6

1.2.1 School Outcomes 6

1.2.1.1 To measure differences (11 PCV vs. placebo) in dropout rates for primary school and secondary school 6

1.2.1.2 To measure differences in permanent dropout rates among children with hearing and language problems.......... 6

1.2.1.3 To measure differences in grade retention for primary schooling and secondary schooling 6

1.2.1.4 To measure differences in school attendance rates for primary school and secondary school 6

1.2.2 Cognitive Outcomes 6

1.2.2.1 To measure differences in IQ as measured by a translated and modified version of the Wechsler Intelligence Scale for Children-Fifth Edition (WISC-V) 6

1.2.2.2 To measure differences in language skills measured by two translated and modified subtests from the Clinical Examination of Language Fundamentals - Fifth Edition (CELF-5)6

1.2.2.3 To measure differences in verbal learning and memory skills measured by three translated and modified tasks from the Wide Range Assessment of Memory and Learning – Second Edition (WRAML-2).......... 6

1.2.2.4 To measure differences in mood or behavioral disturbances assessed via a translated and modified version of the Achenbach System of Empirically-Based Assessment, which includes a self-report form (Youth Self Report) and parent report form (Child Behavior Checklist) ... 7

1.2.2.5 To measure differences in day-to-day problems in aspects of executive functioning, assessed via a translated and modified version of the Behavior Rating Inventory of Executive Functioning – Second Edition (BRIEF-2), both via self and parent report 7

1.2.3 Hearing and otoscopic related outcomes 7

1.2.3.1 To measure differences in rates of otitis media with effusion (OME); adhesive otitis media (AOM); active (draining) chronic suppurative otitis media (CSOM) , inactive (dry perforation, healed perforation) CSOM, and overall CSOM 7

1.2.3.2 To measure differences in rates of mild to moderate and severe hearing impairment (specifically conductive and mixed) in any ear 7

1.2.3.3 To measure differences in rates of OME/CSOM attributable disabling and non-disabling hearing impairment in the best ear 7

Background 7

2.1 Rationale 7

2.1.1 Burden of Otitis Media Sequelae 7

2.1.2 Preventing Otitis Media and Its Sequelae 8

2.1.3 Effects of Otitis Media on Speech and Language Development 8

2.1.4 Effect of Otitis Media Sequela (Hearing impairment, speech and language delays) on School Performance 10

2.2 Conceptual Framework 12

2.3 Hypothesis........ 13

Study Design 13

3.1 Main Trial 13

3. 2 Objectives........ 15

3.2.1 Outcomes to be measured 16

3.2.1.1 Schooling Outcomes........ 16

3.2.1.2 Cognitive Outcomes 16

3.2.1.3 Non-Cognitive outcomes........ 16

3.2.1.4 Additional Family and Child Characteristics........ 17

3.3 Study Design and Conduct 18

3.3.1 Preparatory Steps 18

3.3.2 Obtaining Informed consent. (*Takes place at subjects' home*) 21

3.3.3 School Follow-up for School performance and Attendance records (*at individual schools*) 24

3.3.4 Ear Examinations and Hearing Testing (*at high schools or Barangay Health Centers for dropouts)* 26

3.3.5 Conducting Full IQ Testing, Speech and Language, and Memory testing, as well as evaluation of mood and behavior and day-to-day executive functioning skills (*at high schools or Barangay Health Centers for dropouts*) 28

3.3.6 Obtain Household Socio-economic and Demographic Information 35

3.4 Inclusion and Exclusion Criteria 36

3.5 Study Definitions........ 36

3.5.1 Definition of Otoscopic findings 36

Normal - No evidence of ear disease........ 37

Mild Ear Disease 37
Moderate Ear Disease 37
Severe Ear Disease 38
3.5.2 Audiologic Evaluation 38
Data Quality Control/Quality Assurance Measures 41
4.1 Overall Study Data Quality Assurance- Personnel Roles 41
4.2. Data Quality of Ear Disease Diagnoses 42
4.3. Data Entry Quality Control 43
Statistical Methods 46
5.1 Sample size and power of study 46
5.2 Plan for handling missing data 46
5.3 Main trial endpoints 47
5.3.1 Demographic profile and evaluating balance between treatment groups 47
5.3.2 Evaluating balance between vaccine groups 48
5.3.3 Study endpoints 49
5.3.3.1. The primary analysis 49
5.3.3.2 Secondary analyses 49
5.3.4 Modeling methods 54
5.3.4.1 The primary analysis (school outcomes) 55
5.3.4.2 Secondary analysis 57
5.4 Subgroup/Stratified Analysis 61
References 63

Study objectives

1.1 Primary objectives

1.1.1 To measure differences in academic performance between 11 PCV and placebo groups.

1.1.2 To measure differences in permanent school dropout rates between 11 PCV and placebo groups.

1.2 Secondary objectives

1.2.1 School Outcomes

1.2.1.1 To measure differences (11 PCV vs. placebo) in dropout rates for primary school and secondary school

1.2.1.2 To measure differences in permanent dropout rates among children with hearing and language problems

1.2.1.3 To measure differences in grade retention for primary schooling and secondary schooling

1.2.1.4 To measure differences in school attendance rates for primary school and secondary school

1.2.2 Cognitive Outcomes

1.2.2.1 To measure differences in IQ as measured by a translated and modified version of the Wechsler Intelligence Scale for Children-Fifth Edition (WISC-V)

1.2.2.2 To measure differences in language skills measured by two translated and modified subtests from the Clinical Examination of Language Fundamentals - Fifth Edition (CELF-5)

1.2.2.3 To measure differences in verbal learning and memory skills measured by three translated and modified tasks from the Wide Range Assessment of Memory and Learning – Second Edition (WRAML-2)

1.2.2.4 To measure differences in mood or behavioral disturbances assessed via a translated and modified version of the Achenbach System of Empirically-Based Assessment, which includes a self-report form (Youth Self Report) and parent report form (Child Behavior Checklist)

1.2.2.5 To measure differences in day-to-day problems in aspects of executive functioning, assessed via a translated and modified version of the Behavior Rating Inventory of Executive Functioning – Second Edition (BRIEF-2), both via self and parent report

1.2.3 Hearing and otoscopic related outcomes

1.2.3.1 To measure differences in rates of otitis media with effusion (OME); adhesive otitis media (AOM); active (draining) chronic suppurative otitis media (CSOM) , inactive (dry perforation, healed perforation) CSOM, and overall CSOM

1.2.3.2 To measure differences in rates of mild to moderate and severe hearing impairment (specifically conductive and mixed) in any ear

1.2.3.3 To measure differences in rates of OME/CSOM attributable disabling and non-disabling hearing impairment in the best ear

Background

2.1 Rationale

2.1.1 Burden of Otitis Media Sequelae

The WHO estimates in 2018 that around 486 million people worldwide have disabling HL and 34 million of these are children.[1] In children 60% are due to preventable causes. Over 90% of the burden is borne by the developing world, mainly the countries in the Southeast Asia, the Western Pacific regions, and Africa.[2] Deafness and hearing impairment are major forms of disability in developing countries that cause enormous social, educational and vocational problems.[2] Chronic middle

ear infection is the main cause of mild to moderate hearing impairment in children.[3] Middle ear diseases are a common cause of preventable hearing loss, and chronic suppurative otitis media (CSOM) is the most common cause in developing countries. CSOM, otitis media with effusion (OME), scarring of tympanic membranes and hearing loss are sequelae of acute otitis media (AOM).[4,5] Long-term middle ear effects may include tympanic membrane perforation, ossicular erosion, and cholesteatoma; inner ear effects may include sensory-neural hearing loss, especially for higher frequencies.[6] Since CSOM is most likely to follow episodes of either incompletely or inadequately treated bacterial AOM the problem of under recognized AOM could have a direct impact on the burden of chronic otitis media and its sequelae.[7]

2.1.2 Preventing Otitis Media and Its Sequelae

An 11-valent pneumococcal vaccine,[8] and more recently a 10-valent pneumococcal vaccine (PCV), that uses protein D of *Haemophilus influenzae* (PHiD-CV) as a protein carrier have been shown to prevent acute otitis media caused by both *S. pneumoniae* and nontypeable *H. influenzae*.[9-12] PCV 7 and 13 have also been shown to reduce AOM in industrialized countries.[13] However there are no randomized controlled trials of a PCV in developing countries that examine the long-term impact of PCV on the prevention of OME/CSOM and its sequelae, including the potential impact on speech and language development, overall thinking skills, or school performance and future earning potential.

2.1.3 Effects of Otitis Media on Speech and Language Development

There is abundant research that outlines the effects of hearing loss on speech and language development. The younger the age of onset of hearing difficulty, the greater the impact on normal development, especially when presented with structurally-complex language, potentially due to difficulty with acquisition and processing of syntactic information.[14] The American Speech-Language Hearing association identifies four areas of impact of hearing loss: [15]

1. Delayed development of receptive and expressive communication skills (speech and language).
2. Language deficit causing learning problems that result in reduced academic achievement.
3. Communication difficulties often leading to social isolation and poor self-concept.
4. Vocational choices.

Children with hearing loss have delays in vocabulary acquisition, particularly abstract vocabulary. This is compounded by the fact that developing children typically acquire vocabulary through oral language (listening to others and speaking with others) through about third grade. Beyond the third grade, developing children acquire vocabulary through exposure to text. For children with hearing loss, not only is the oral language route to vocabulary fraught with roadblocks, but also reading is virtually always impacted and therefore, children with hearing loss face a series of cascading consequences in vocabulary development. In the reading literature, this is known as the Matthew Effect.[16] Learning to read is difficult for this population because reading acquisition is based on the phonological or sound system of a child's language. Since the phonological system is less than adequate, reading skills are not developed at a typical rate. Without the vocabulary to support decoding or sounding out words, hearing impaired readers struggle with comprehension. When comprehension is compromised, readers don't enjoy reading and typically choose to read less and less. Since hearing impaired readers are reading fewer words, their vocabulary stagnates resulting in even less desire to read. As such, hearing loss and the resulting speech and language deficits promulgate a cascade of events that impact academic school performance, which is a consistent finding in the literature in terms of delay in acquisition of early reading skills.[17]

2.1.4 Effect of Otitis Media Sequela (Hearing impairment, speech and language delays) on School Performance

Children with mild to moderate hearing loss achieve one to four grades lower than their typically developing peers, and those with severe to profound hearing loss experience a delay in acquisition of early reading skills,[17] and rarely achieve reading skills greater than the third grade level,[15] and reading skills are imperative to broader academic achievement throughout childhood. Studies indicate that children with hearing loss have significantly lower scores on academic tests of word recognition, spelling and language. Such problems lead to greater need for special education, greater likelihood of repeating a grade, and higher dropout rates.[18-20] Children with hearing loss are more likely to fail entrance exams for secondary education (e.g., advanced high-school, college).[21] Many of these studies have been carried out in high-income countries; there is, in fact, remarkably little research examining the academic achievement of hearing impaired children in low- or middle-income countries. The long-term social and economic impacts of low literacy skills and poor school achievement cannot be underestimated. Children with less formal schooling have worse labor market prospects and higher unemployment. There is also sufficient evidence that lower literacy skills are highly correlated with the numbers of people involved in the criminal justice system.[22] Given these outcomes, understanding the mechanisms by which young adults with hearing loss have difficulty achieving economic stability, could be potentially enormous.

Difficulties in academic achievement suggest that otitis media may have a negative effect on overall cognitive capacity and thinking skills, such as intellectual ability (IQ) and especially verbal skills. Several large cohort studies have assessed for this association in infancy/toddlerhood, young childhood, and in middle school children; however, to our knowledge, none have assessed for impact of childhood otitis media on intelligence in teenage years, which is a critical time point during which children may be selecting careers or making employment or additional schooling plans once they graduate. Results in the younger cohorts have been mixed. While several studies report limited association,[23-25] far more have

found positive associations in that increased time spent with otitis media or the more severe intervention required for treatment was related to decreased IQ scores.[17,26-31] In addition, adults who had been hospitalized with otitis media in early childhood are reported to have lower IQ scores.[32] These discrepant results have prompted discussion of potential impact of various methodological (i.e., inadequate documentation of otitis media, differing otitis media outcome variables, sample sizes too small to detect moderate effects, poor assessment tool sensitivity, and use of different estimators to measure IQ) issues. Due to our robust sample of participants, our group is uniquely positioned to investigate the relationship between otitis media and IQ in teenagers. Not only will we have a significantly large sample size, but otitis media was well documented and characterized when they were young. Lastly, we will estimate IQ using the gold standard measure of IQ in children, the Wechsler Scales, which have already been shown to be sensitive to intellectual differences in youngsters with a history of otitis media.[28] We will also conduct a full assessment instead of prorating or estimating from only administering certain individual subtests and interpret all scores obtained in this assessment (i.e., verbal intellectual skills), not simply overall IQ.

In addition to intelligence, other factors may better explain school difficulties in these children. Potentially secondary to speech and language deficits, children with otitis media also have reduced verbal working memory,[33,34] such that the amount of verbal information they are able to keep and hold in mind is reduced. These children may therefore be at risk for difficulties acquiring verbal information and remembering it accurately over time. This relationship has not been widely investigated, though Dewey and Wall[35] did find memory deficits in language-impaired children. Further clarification of the memory abilities of children with otitis media is therefore warranted, especially as much of academic learning is verbally-based. Better understanding of learning and memory profiles of these children will further elucidate the real-world impact of language deficits secondary to otitis media. We plan to investigate the memory profiles of participants using several tasks from the Wide Range Assessment of Memory and

Learning (WRAML-2),[36] which is a well-validated and highly used measure of memory functioning in children and adults.

Certain socioeconomic and behavioral characteristics of children with otitis media have also been reported to explain a significant portion of variance in overall thinking skills and developmental outcome;[25,29,30] inadequate control of such environmental confounders has been discussed as potential contributors to non-significant findings in the literature. As such, acquiring ratings on effects such as socioeconomic status, attention, and anxiety will be important to determine their relative contribution to performance on standardized measures. In our cohort, we will be able to acquire parent and self-reported ratings, which will provide increasingly reliable data. We plan to use the Achenbach System of Empirically-Based Assessment[37] to measure any mood or behavioral disturbances, which include self and parent report forms. We will also measure executive functioning skills, which can greatly interfere with academic success.[38] Together, these measures provide a thorough estimate of behavioral and psychiatric indicators that may play a prominent role in explaining additional reasons for differing levels of academic achievement.

Altogether, the combination of assessments of IQ, memory, executive function, and mood paired with a comprehensive oral language assessment is expected to include or exclude the underlying causes of children's poor educational outcomes.

2.2 Conceptual Framework

Figure 1 shows the conceptual framework guiding this study. It shows the primary and intermediate pathways by which PCV 11 vaccination affects hearing, cognition, behavior and educational success.

Figure 1. Study conceptual framework

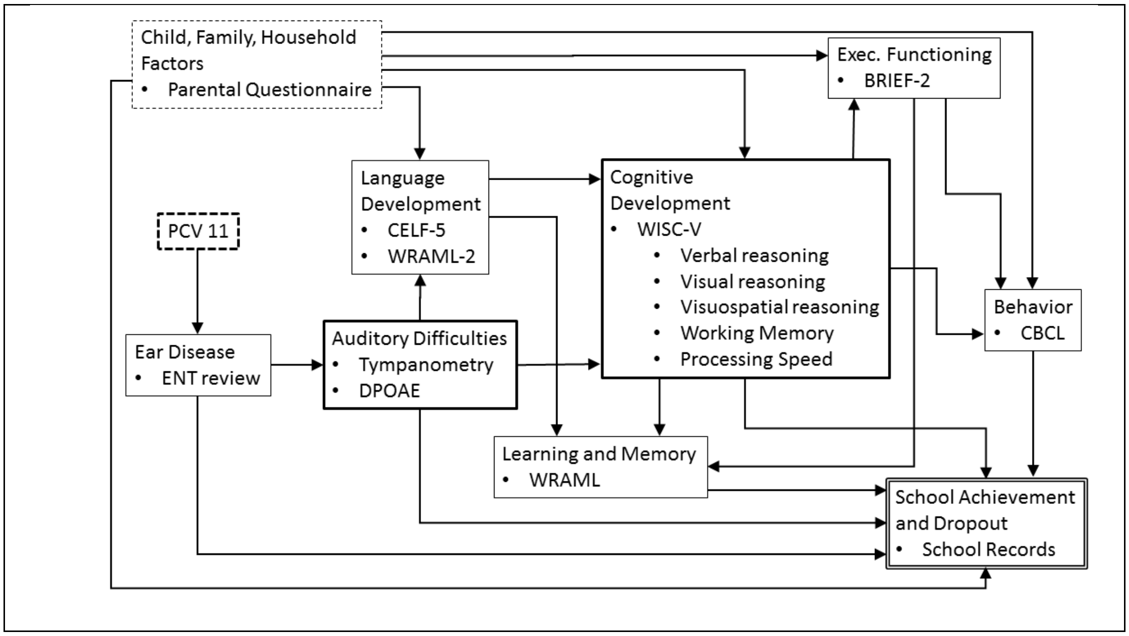

2.3 Hypothesis

The study hypothesis is that the prevalence of otitis media with effusion (OME), chronic suppurative otitis media (CSOM) and hearing impairment arising from OME and CSOM is reduced in children vaccinated with 11 PCV compared to controls. As such, preventing OME and CSOM, results in better hearing and more advantageous speech and language development in the 11 PCV vaccinated children, leading to improved school performance.

Study Design

3.1 Main Trial

In 2000 to 2004, we conducted a randomized, placebo-controlled, double-blind trial in Bohol, the Philippines.[39] Children 6 weeks to <6 months of age were randomly allocated to receive 3 doses of either an 11-valent PCV (11PCV, sanofi pasteur, Lyon, France) or a saline placebo, with a minimum interval of 4 weeks between doses to determine vaccine efficacy (VE) against the primary outcome of a child

experiencing first episode of community-acquired radiologically defined pneumonia in the first 2 years of life. Secondary end points were clinical pneumonia, invasive pneumococcal disease, safety, and immunogenicity. In per-protocol analysis, a 22.9% reduction of community-acquired radiologically confirmed pneumonia in children younger than 2 years of age in the 11PCV vaccinated group was observed; a reduction similar as observed in other PCV trials. We could not demonstrate any VE against clinical pneumonia.

Block randomization was used to allocate 11PCV and placebo vaccines at the individual level. A list containing random permutations of the letters A to F was generated by sanofi pasteur using SAS software (SAS Institute, Inc, Cary, NC); 3 of the letters were allocated to the 11PCV vaccine and 3 to the placebo. The correspondence

between letters and vaccine type was unknown to researchers or subjects. After a child was enrolled, he or she was allocated to the next available letter on the list. Vaccine letter codes were concealed from the study nurse until uncovered at the time of allocation. The corresponding lettered solution was administered to the infant and recorded on the case report form. The code list was kept in sealed envelopes at 3 sites only: sanofi pasteur, the safety monitor of DSMB, and the Independent Technical Services Unit located at the National Health and Medical Research Council Clinical Trials Centre, based at the University of Sydney (Australia).

Subsequent to the main trial, we conducted a 10 to 15 year follow-up of the 12,000 children enrolled in a randomized placebo-controlled trial of an 11-valent pneumococcal vaccine conducted between 2000 and 2004 in Bohol, Philippines.[39] The intent of was to follow these children to determine differences between the two groups of children [vaccinated and placebo groups] in the sequelae of otitis

media, the impact on hearing, and its subsequent impact on speech and language development, intelligence, cognitive and behavioral functioning, and school performance. Given the large sample size, we approached it in a stepwise manner.

1. **Obtain informed consent** from study participants in their homes, administer a household socio-demographic survey which included a consumption and employment module, collect available school records, and identify whether the participants are still in school, are working, or have died in the interim.
2. **Follow-up in the schools**, after obtaining permission from the Department of Education, and in conjunction with the school district, obtain school records of class examinations, and results of national school examinations, by subject.
3. **Conduct ear and hearing exams** using video-otoscopy, and tympanometry, and conducting a hearing exam for all study children at their respective schools; and at the Barangay Health Center, if dropped out but still traceable.
4. **Conduct neuropsychological testing**, including assessment of IQ as well as language, memory, executive functioning, and mood on all subjects (e.g., all children who dropped out and/or had an abnormality on ear or hearing exams).

3. 2 Objectives

The objective of this study was to measure the effect of the 11 PCV on hearing, cognitive functioning and school performance.

To reach this objective we compared outcomes in 11 PCV vaccinated subjects and controls, 12 to 18 years after randomization to the vaccine or placebo groups. We will control for possible confounders including information on household demographics and baseline socio-economic status to the extent we are able. In addition, we will examine if the treatment effects differ on important

dimensions, including sex, demographics, and distance to important institutions such as health care clinics or school.

3.2.1 Outcomes to be measured

3.2.1.1 Schooling Outcomes

1. School dropout and attendance rates.
2. Academic performance as measured by school marks. Note that these may be biased since they are only on people who were in school.

3.2.1.2 Cognitive Outcomes

1. IQ as measured by the Wechsler Intelligence Scale for Children-Fifth Edition (WISC-V).[40]
2. Language skills using two tasks from the Clinical Examination of Language Fundamentals - Fifth Edition (CELF-5).[14]
3. Memory abilities using several tasks from the Wide Range Assessment of Memory and Learning (WRAML-2).[36]
4. Mood or behavioral disturbances, using the Achenbach System of Empirically-Based Assessment (both self-report [YSR] and parent report [CBCL]).[37]
5. Day-to-day executive functioning, using the Behavior Rating Inventory of Executive Functioning – Second Edition (BRIEF-2).[38]

3.2.1.3 Non-Cognitive outcomes

1. Rates of OME and other types of mild and moderate ear disease, active, inactive and overall CSOM.
2. Rates of mild to moderate and severe hearing impairment.
3. Rates of OME/CSOM attributable disabling and non-disabling hearing impairment.

3.2.1.4 Additional Family and Child Characteristics

1. Household socio-demographic and environmental characteristics that may contribute to cognition, language development, school performance, and health (e.g., parental education and occupation, household income and consumption, family structure, presence of parents, number/age of siblings, etc.)
2. Self-reported measures of health (chronic morbidities such as asthma) to determine how the vaccine affects health.
3. Measurements of temperament and soft skills.
4. Preschool attendance and school attendance.

3.3 Study Design and Conduct

This was a follow-up study of the subjects recruited into the ARIVAC trial between 2000 and 2004. The ages of children from the ARIVAC at study start are illustrated in **Table 1.**

Table 1. Children's Age as of 2016 at start of the study By Municipality, Who Participated in the ARIVAC study 2000 to 2004

Municipality	Age (in years)					
	11	12	13	14	15	Total
**TAGBILARAN**	310	1511	1731	1744	505	5801
**DAUIS**	107	611	638	660	192	2208
**PANGLAO**	76	385	434	385	153	1433
**BACLAYON**	52	234	221	235	76	818
**CORTES**	35	262	247	268	75	887
**BALILIHAN**	55	305	267	331	89	1047
**Total**	635	3308	3538	3623	1090	12194

3.3.1 Preparatory Steps

1. Obtain list of all ARIVAC participants. Sources of data: Existing ARIVAC dataset obtained from ARIVAC data managers in Bohol and Manila
2. Obtain addresses of ARIVAC participants who were found in the GPS study of 2009-10.
3. The age of the participants was calculated using the Date of Birth and the date of the start of the current study. Age in years was arranged from oldest to youngest.

4. Simultaneous activity: Letters were written to Municipal Mayors, Municipal Health Officers, Barangay Health Captains in the relevant municipalities and barangays in Bohol asking permission to visit them to discuss the objectives of the study and the importance of doing this study.
5. Simultaneous activity: Visits to the Department of Education office in Tagbilaran City, Bohol to discuss the objectives of the study and the importance of conducting the study. We requested the permission of the Department of Education to obtain the school records of all ARIVAC children if the parents of the children would allow us to do this in case the family of these children have lost/misplaced the school records of the children. School records were defined as the school cards of the children containing the grades of the school children.

Department of Education Clearance: To ensure that, with the informed assent/parental consent of research participants and parents, we would have access to school records, we contacted and networked with the DepEd Officials at the regional, provincial/city, district and municipality levels. A stepwise approach was employed in obtaining approvals and endorsements for school records access, as follows

A) Constructing a directory of school officials at all levels and principals of all elementary and high schools where the earlier vaccine study was conducted;

B) Requesting a meeting, first, at the regional level for the purpose of (1) orienting them about the study and the need to access school records, (2) requesting information on the steps to be employed in obtaining permission to access school records, whether for public or private schools. This study complied with the necessary procedures to obtain approval. The final output was an endorsement from the DepEd National or Regional

Officials to the DepEd Provincial and City officials to allow the study access to the school records..

C) The endorsement from the DepEd National and Regional Officials were then carried down to the Tagbilaran City and Bohol Provincial Superintendents of Schools. The Tagbilaran City Superintendent's Office has authority over all the elementary and secondary schools located within the city; while the Bohol Provincial Superintendent's Office has authority over all the public and secondary schools in the province. Because of the breadth of the administrative function of the Provincial Superintendent's office, the province is divided into districts. Each district oversees and supervises several municipalities or towns depending on the number of schools in each. Since this study followed up the participants in 11-valent Pneumococcal Vaccine Trial that was conducted in one city and five municipalities, we needed to coordinate with at least three districts under the Provincial Superintendent's Office. From both the Tagbilaran City and Provincial Superintendent's Offices, we obtained the authorization to access the school records of the study participants for as long as we had the informed assent and parental consents.

D) Once endorsements were acquired down to the level of the principal in each of the elementary and secondary schools, we conducted orientation meetings for the following purposes: (1) orient them about the pneumococcal vaccine trial and the current study, (2) the tests which will be conducted on selected students, their risks and benefits, and (3) the assistance we were requesting from them in the conduct of these procedures.

Department of Health (DoH) Clearance:: Since it was some time since the study had been conducted on the island, we also needed to reconnect with the DOH officials to obtain the necessary approvals and endorsements from the Central or Regional Offices to (1) request

assistance from the Midwives and Barangay Health Stations to locate the earlier trial participants and (2) to conduct the necessary tests on the children either at the City or Rural Health Units or Barangay Health Stations. We observed the procedures A and B as indicated above. However, the endorsements we obtained from the National and Regional offices to the City and Provincial Health Officers, were regarded as simply endorsements. The health services in the country have devolved since 1995, and as such health workers are under the administrative responsibility of their respective local government units headed by the City or Municipality/Town Mayors. We counted on the continued good relationship we had maintained with the research sites for us to acquire their assistance and cooperation.

Once the endorsements were received at the City and Municipal Health Offices level, we conducted orientation meetings with the health staff. We conducted orientation meetings for the following purposes: (1) orient them about the pneumococcal vaccine trial and the current study, (2) the tests which will be conducted on selected students, their risks and benefits, and (3) the assistance we will be requesting from them in the conduct of these procedures.

3.3.2 Obtaining Informed consent. (*Takes place at subjects' home*)

In 2008-2010, we conducted a follow-up survey of all children recruited to the ARIVAC study, using addresses and GPS, to map every household.[41] We were able to identify over 90% of all households during that survey, and have subsequently completed analyses of this data, linking the households with subjects in the trial.[42,43] We now have definitive identification of the households where the majority of our initial subjects resided, most of whom currently reside in the same houses.

On Bohol Island, school records of children, for every grade, are collected by the schools themselves at the beginning of the New Year. The overall performance over the year for each of the subjects is summarized in the school record. In addition, school attendance is marked on a monthly basis. In order for us to obtain access to the school records, we needed to obtain informed consent from the parents or guardians prior to contacting the school.

Study participants were contacted by barangay, beginning with the most urban barangays where the majority of study children live, and then moving to rural areas. Rented cars and motorcycles were used to transport research associates to the houses of the ARIVAC children. When possible, older children were tracked first before they graduated from secondary school and became difficult to track. When all participants in a barangay were found and information collected, we proceeded to the next barangay.

Upon reaching the houses, trackers introduced themselves, then ask about:

a. The whereabouts of the ARIVAC participant (using the name)
b. If the participant was still living in the house.
c. If the parents of participants are living in the same household
d. Permission for the research associates to tell the parents about the study
e. Obtaining informed consent

If the participant was not living in the same house where he/she used to live during the GPS study, the research associates then inquired as to where the participant or his/her family were residing currently. If the information was available, the tracker noted these details, and a mobile phone number if available, and scheduled a visit to the village where the participant was currently residing. Additional questions might refer to whether the participant ever went to school and if the current householders know the school where the participant studied.

If the information was not available to the current participant, other nearby households or the Barangay Health Centers were approached to ask the whereabouts of the family. For participants who were tracked to places outside the study area, we either scheduled a visit with the participant if they were located in an area study staff can visit, or if they were able to complete a short phone interview.

After obtaining the informed consent for their children to participate in the study, the trackers collected more information on the following:

a. Whether participant studied or not
b. If participant studied, history of schools where the participant studied
c. Availability of school records (cards) of the participant from elementary grade through high school.
d. If school records are available, we asked permission from the parents to get a picture of the school records (serves as a copy of the school records)
e. Copies of the school records extending as far back as Grade 1 up to the last school year attended by the participant were obtained.
f. If school records were not available, parents signed a document authorizing the study nurses to obtain a copy of the school records from the schools where the participant studied.
g. Inquire whether the participant ever dropped out of school and when this occurred. Inquire as to the reason for dropping out of school.
h. Household socio-demographic characteristics through a short survey of the head of household.
i. GPS data on location of the household.

3.3.3 School Follow-up for School performance and Attendance records (*at individual schools*)

Occurring simultaneously with the process of obtaining IRB approval, we contacted the Department of Education, Bohol Island, to obtain approval to approach all of the elementary schools and high schools in the 48 Barangays in six municipalities, where subjects were recruited for the original study. Each Barangay has one elementary school, and each municipality has between two and six or more high schools **(Table 2).** Since our subjects would all have gone to elementary schools in their own Barangays, we needed to approach the headmasters of all of the elementary and high schools, after obtaining informed consent from all of the subjects' parents, to access their school performance records from kindergarten/first grade all the way through 12th grade. National exams are held in sixth grade and upon graduation. Individual level results were obtained for all children who had completed sixth grade.

Table 2. Number of Schools in the ARIVAC Study Municipalities

Municipality	Elementary Schools		Secondary Schools	
	Public	Private	Public	Private
Tagbilaran	16	16	6	13
Baclayon	12	1	2	1
Balilihan	17	0	3	1
Cortes	12	0	2	1
Dauis	12	1	3	1
Panglao	12	1	1	3
Total	81	19	17	20

Study personnel scheduled the visits at the schools where the participant studied. A letter was written to the school principal together with the approval letter from the Director of the Department of Education located in Tagbilaran City and the authorization letter from the parents to obtain the school records of the participants from Grade 1 up to the present, if applicable. They then collected the school records of the participants, and scanned them in for data entry. GPS locations of schools were collected at this time.

3.3.4 Ear Examinations and Hearing Testing (*at high schools or Barangay Health Centers for dropouts)*

Enrollment and flow of study[44]: Briefly, we had three groups of study nurses that worked with each classroom of 40-50 students.

a. Trained nurses (Group 1) did otoscopic screening of subjects using otoscopes with illuminated heads (Welch-Allyn, Skaneateles Falls, NY, USA) and checked for persistent cleft palate, repaired cleft palate and sub mucus as potential confounders
b. Trained Nurses (Group 2) removed wax with wax curettes and performed the tympanometry and distortion product otoacoustic emissions (DPOAEs) tests with the Sentiero Desktop Screening OAE w/ Screening Tympanometer (Path Health, Germany).
c. Trained nurses (Group 3) did the audiology screen of subjects in quiet rooms, using the hearScreen Samsung Galaxy J2 Smartphone and ISO Calibrated Noise Cancelling Sennheiser HD280-pro Circumural Headphones (0.5- 8kHz), with hearScreen and hearTest software (mHealth Studio, Pretoria, South Africa). We did this as follows:

Otoscopic Examination: Trained nurses screened all students, who already had consent forms and questionnaires completed by their parents/caregivers. Those children with wax in the ear canals had wax removal done by trained nurses who will also do tympanometry/DPOAE and free field hearing screening. If the ear drum was obstructed by wax or a foreign object that could not be removed, the child was referred to an otolaryngologist (i.e., ENT specialist) and asked to reschedule for their exam. The child was also referred to an ENT if they had otorrhea, which indicates a current infection, and were asked to reschedule when the ears are clear. We used video-otoscopy to record all otoscopic findings, electronically. This data was downloaded to the database daily, and linked to the subjects, by subject ID.

Tympanometry: Tympanometry measures the efficiency of the middle ear system (tympanic membrane and ossicular chain) in conducting sound from the external, air-filled environment to the internal, fluid-filled environment (the inner ear). Residual effects of otitis media in childhood, such as ossicular chain erosion, tympanic membrane perforation, and/or tympanosclerosis, may be detected by this measure. Tympanometry was conducted in a quiet environment. A sound treated booth was not required to conduct this measurement. Tympanometry data were saved electronically, linked to the subjects and uploaded to a Redcap database[45] daily.

Distortion product otoacoustic emissions (DPOAEs): Extended high-frequency distortion product otoacoustic emissions measure the response of sensory cells in the inner ear to acoustic stimulation. Inner ear sensory cells that have been damaged will be detected by this measure. DPOAEs were conducted in a quiet environment. A sound treated booth was not required to conduct this measurement. This data was collected along with the tympanometry data and saved electronically.

Audiologic screening and evaluation: All children received audiology screening to identify possible hearing loss. Audiologic screening was conducted in a quiet environment under noise-attenuating earphones. Children who met criteria for possible or probable hearing loss during the audiology screen, as well as any of the following criteria, were referred to the Bohol Hearing Center (BHC) for a formal audiology evaluation:

1. a history of ear discharge, ear drum perforation, or hearing problem
2. an abnormal tympanogram or

3. a failed DPOAE test.

Audiologic evaluation measures the child's response to the softest sound he/she can hear at octave frequencies from 500 Hz through 8,000 Hz. This measure quantifies the degree of hearing loss in each ear individually, and classifies the type of hearing loss as conductive, sensorineural, or mixed.

Otolaryngologist Review: Video-oscopic data, tympanometry, DPOAE and audiometry data were stored in the RedCap database. An otolaryngologist will review the video-otoscopy for each ear on a patient-by-patient basis and classify the signs of ear disease, using the definitions described later. The signs and symptoms of ear disease will be used to classify the type and severity of ear disease (see page 34). Ear canal and/or tympanic membrane and audiometric abnormalities will be reviewed by an otolaryngologist in Bohol, and will be used to determine required follow-up care.

3.3.5 Conducting Full IQ Testing, Speech and Language, and Memory testing, as well as evaluation of mood and behavior and day-to-day executive functioning skills (*at high schools or Barangay Health Centers for dropouts*)

All children will undergo a cognitive assessment lasting approximately 1.5 hours to determine strengths and weaknesses in cognitive functioning in the domains of intellectual functioning (e.g., verbal, visual, and visuospatial reasoning, working memory, processing speed) using the Wechsler Intelligence Scale for Children- Fifth Edition (WISC-V), which is the gold-standard measure of IQ in children[40], language comprehension using selected subtests from the Clinical Evaluation of Language Functions-5 (CELF-5), and verbal and visual learning and memory using tasks from the Wide Range Assessment of Memory and Learning-2 (WRAML-2). In addition, participants and their parents will

endorse any problems in aspects of mood/behavior and executive functioning on formal questionnaires. For mood/behavior, we will use the Achenbach System of Empirically-Based Assessment[37] which includes a self-report (the Youth Self Report) and parent report (Child Behavior Checklist) forms. To determine day-to-day executive functioning, we plan to use the Behavior Rating Inventory of Executive Functioning – Second Edition (BRIEF-2, again self and parent forms). Study staff will be trained to administer the assessments by the neuropsychologist and will administer these tests. Testing will be completed at high schools, in a batched manner, starting with the oldest children. For children who have dropped out of school, since we would have obtained consent from the parents, we will administer the tests at the Barangay health centers.

Intellectual Functioning: WISC-V: *Similarities, Vocabulary, Block Design, Matrix Reasoning, Figure Weights, Digit Span, Coding subtests*. The WISC-V is the most current measure of the Wechsler Scales, which were first published over 70 years ago. Norms were collected during April 2013 to March 2014 from a sample stratified on key demographic variables according to 2012 US Census data. There are numerous published studies of convergent and discriminant validity, construct validity, as well as clinical utility. Average subtest reliability coefficients range from good to excellent, and all subtests possess adequate stability in test/re-test over time. Overall intellectual functioning will be determined from performance on the 7 subtests listed above. Vocabulary and Similarities comprise the *Verbal Comprehension Index*, which is a measure of core language skills. The Vocabulary subtest assesses word knowledge and verbal concept formation and is measured by having participants define words. The Similarities subtest assesses verbal concept formation and abstract reasoning and is measured by having participants describe how two given words are similar. Both subtests are untimed. The *Visual Spatial Index* is a measure of core visuospatial abilities. Individuals only completed one subtest from this Index

(Block Design). Block Design measures the ability to analyze and synthesize abstract visual stimuli and is assessed by having participants assemble blocks to look like pictures that they view under time pressure. The *Fluid Reasoning Index* is a measure of nonverbal abstract reasoning abilities. The Index is comprised of two subtests (Matrix Reasoning and Figure Weights). Matrix Reasoning assesses visual classification and knowledge of part-whole relationships and is measured by having participants select the item that completes an incomplete matrix or series of pictures/designs. This subtest is untimed. Figure Weights measures the quantitative concept of equality and the application of the concepts of matching, addition, and/or multiplication. Under time pressure, participants view a scale with a missing weight and select a response that keeps the scales balanced. The *Working Memory Index* is a measure of working memory (e.g., the ability to hold information "in mind." Individuals completed only one subtest from this Index (Digit Span). Digit Span is divided further into 3 tasks, including Digit Span Forward, Digit Span Backward, and Digit Span Sequencing. All tasks require focused attention and auditory rehearsal. During Digit Span forward, participants hear a series of increasing sequences of numbers and repeat them back in the same order, which measures temporal storage capacity in working memory. During Digit Span Backward, participants again hear increasing sequences of numbers and they repeat them back in reverse order, which measures mental manipulation and transformation of information via maintaining the location of each number in the series. During Digit Span Sequencing, participants again hear increasing sequences of numbers and they repeat them back in numerical order from lowest to highest, which measures mental manipulation via ability to maintain the quantitative value of the number. Lastly, the Processing Speed Index is a measure of cognitive and graphomotor processing speed. Individuals completed only one subtest from this Index (Coding). Coding measures psychomotor speed, visual-motor coordination, and visual scanning. Under time pressure, participants use a key to copy symbols that correspond to specific numbers.

Language assessment: *CELF-5 Following Directions and Semantic Relationships subtests as well as WRAML Sentence Memory*. These subtests will be used to gain knowledge of the language skills of all children in the study. Although normed in the United States, CELF scoring is sensitive to various dialects and diverse cultures. The Following Directions subtest is a measure of one's ability to interpret spoken directions of increasing length and complexity, which is an essential skill for the classroom and at home. The Semantic Relationships subtest evaluates the ability to interpret sentences that make comparisons, identify location or direction, specify time relationships, include serial order, or are expressed in passive voice, which is required when interpreting conceptual relationships, which is a common occurrence in any classroom curriculum or social setting. Sentence Memory is a measure of auditory working memory where participants are presented with increasingly complex sentences only a single time that they are required to repeat.

Memory Assessment: *Story Memory, Verbal Learning, Picture Memory***.** The WRAML-2 is a collection of individual measures designed to assess **memory ability** and is widely used in neuropsychological assessment of children and adolescents (Bigler & Adams, 2001). The WRAML is sensitive to memory deficits in language-impaired children (Dewey & Wall, 1997). The WRAML-2 is comprised of three psychometrically validated factors (i.e., verbal memory index, visual memory index, and attention/concentration index) that can be combined into an overall index of general memory functioning. *Story Memory* is a contextual verbal learning and memory task. Participants are read two short stories of differing developmental levels of interest and linguistic complexity. They are then asked to freely recall as much of the story as they can. After a delay of approximately 15 minutes, participants are asked to again recall as much as they can remember from both stories. After this recall, participants

are asked to discriminate details from the story in a yes/no recognition format. Raw scores are then collected for *Story Memory* that are used to determine scaled scores and overall performance on the task:

a. Story Memory: Total score from initial recall of both stories.
b. Story Memory Delay Recall: Total score from delayed recall of both stories.
c. Story Memory Recognition: Total score from yes/no recognition questions for both stories.
d. Story Memory Retention: Difference between Story Memory Delayed Recall and Story Memory Learning.
e. Story B Memory: Score from initial recall of first story
f. Story C Memory: Score from initial recall of second story
g. Verbatim Measure: Number of verbatim responses for each story administered.
h. Gist Measure: Number of points for recalling the “gist” of each story.

Verbal Learning is a rote verbal list-learning and memory task. Participants are read a list of 16 words over 4 learning trials. After each learning trial, they are asked to recall as many words as they remember in any order. Approximately 10 minutes later, individuals are asked to again recall as many words as they can remember. Approximately 10 additional minutes later, individuals are asked to discriminate in yes/no format words that were on the list from 40 total words. Raw scores are then collected for *Verbal Learning* that are used to determine scaled scores and overall performance on the task:

a. Verbal Learning: Total score from all 4 learning trials.
b. Verbal Learning Delay Recall: Total score from delayed recall trial.
c. Verbal Learning Recognition: Total score from recognition trial.

d. Verbal Learning Retention: Difference between performance on the delay recall trial and trial 4.
e. Verbal Learning Slope: Difference between performance on Trial 4 and Trial 1.
f. Verbal Learning Intrusion Errors: Total number of intrusion errors on 4 learning trials.

Picture Memory is a contextual visual learning and memory task. Participants are shown four common but visually complex scenes for 10 seconds. Then they are shown a similar alternate scene and asked to identify the elements that have moved, changed, or added. Following a delay, individuals are then shown 44 picture elements and asked to indicate if the element was previously seen on one of the four original or alternate scenes. Raw scores are then collected for *Picture Memory* that are used to determine scaled scores and overall performance on the task:

a. Picture Memory: Total score from all 4 scenes.
b. Picture Memory Recognition: Total score from recognition trial.
c. Picture Memory Commission Errors: Total number of errors (items erroneously chosen or false negatives).

Certain socioeconomic and behavioral characteristics of children with otitis media have been reported to explain a significant portion of variance in overall thinking skills and developmental outcome.[25,29,30] As such, acquiring ratings on effects such as socioeconomic status, attention, and anxiety will be important to determine their relative contribution to performance on standardized measures. In our cohort, we will be able to acquire parent and self-reported ratings, using the Achenbach System of Empirically-Based Assessment[37] to measure any mood or behavioral disturbances, which includes a self-report form (the Youth Self Report) and parent report (Child Behavior Checklist). To determine day-to-day executive functioning, we plan to use the Behavior Rating Inventory of Executive Functioning – Second Edition (BRIEF-2).[38] Together, these two measures provide a thorough estimate of behavioral

and psychiatric indicators that may play a prominent role in explaining differing levels of academic achievement. The combination of executive function, IQ, memory, and mood tests paired with a comprehensive oral language assessment is expected to include or exclude the underlying causes of children's poor educational outcomes.

Mood and Behavior: Achenbach System of Empirically-Based Assessment[37] self-report (the *Youth Self Report, YSR*) and parent report (*Child Behavior Checklist, CBCL*) forms. The YSR consists of 112 not true/somewhat true/mostly true questions that ask about problems in the domains of anxiety/depression, withdrawal/depression, somatic complaints, social problems, thought problems, attention problems, rule-breaking behavior, and aggressive behavior over the past 6-months. Responses are summed into each domain. The combination of anxious/depressed, withdrawn/depressed, and somatic complaints can be further summed into Total Internalizing Problems; Rule-Breaking and Aggressive Behavior can be further summed into Externalizing Problems.

The CBCL is the parent version of the YSR and consists of 113 not true/somewhat true/mostly true questions. Like the YSR, the CBCL asks about problems in the domains of anxiety/depression, withdrawal/depression, somatic complaints, social problems, thought problems, attention problems, rule-breaking behavior, and aggressive behavior. Responses are summed into each domain. The combination of anxious/depressed, withdrawn/depressed, and somatic complaints can be further summed into Total Internalizing Problems; Rule-Breaking and Aggressive Behavior can be further summed into Externalizing Problems.

Day-to-day Executive Functioning: Behavior Rating Inventory of Executive Functioning – Second Edition (BRIEF-2). The BRIEF-2 also has self-report and parent forms. The BRIEF-2 self-report form consists of 55 questions in which the participant answers how frequently (never/sometimes/often) the following problems have happened to them over the past 6-months. Responses are them summed into 7 scales: Inhibit, Self-Monitoring, Shift, Emotional Control, Task Completion, Working Memory and Plan/Organize. The summation of Inhibit and Self Monitoring is the Behavior Regulation Index. The summation of Shift and Emotional Control is the Emotion Regulation Index. The summation of Task Completion, Working Memory, and Plan/Organize is the Cognitive Regulation Index. Together, the Behavior Regulation, Emotion Regulation, and Cognitive Regulation Indexes form the Global Executive Composite, an overall measure of problems in day-to-day executive functioning.

The parent report form is similar, consisting of 63 never/sometimes/often questions for the past 6-months. Responses are summed into 9 scales: Inhibit, Self-Monitoring, Shift, Emotional Control, Initiate, Working Memory, Plan/Organize, Task Monitor and Organization of Materials. Again, the summation of Inhibit and Self Monitoring is the Behavior Regulation Index and the summation of Shift and Emotional Control is the Emotion Regulation Index. The Cognitive Regulation Index is the summation of Initiate, Working Memory, Plan/Organize, Task Monitor and Organization of Materials. Together, the Behavior Regulation, Emotion Regulation, and Cognitive Regulation Indexes form the Global Executive Composite, an overall measure of problems in day-to-day executive functioning.

3.3.6 Obtain Household Socio-economic and Demographic Information

Health, educational and cognitive outcomes of children and young adults are highly dependent on household socioeconomic circumstances and the value parents place on childhood experiences such as schooling. During the initial household visit, after informed consent has been obtained, the head of

household will be given a survey to collect data on family member demographics (age, sex, education), household socioeconomic characteristics (e.g., occupation, consumption, perceived socioeconomic status), general health status (e.g., do parents have health problems, do children have chronic health problems), household environment (e.g., type of cooking fuel, type of materials in home), and risk factors (e.g., exposure to household and/or cigarette smoke). These are potentially confounding factors which may also affect the relationship between the vaccine and health, educational, and cognition outcomes. GPS data will also be collected at this time.

3.4 Inclusion and Exclusion Criteria

Inclusion Criteria:

1. Participant in the previous PNF 13 pneumococcal vaccine trial conducted between 2000 and 2004.
2. Signed informed consent.
3. Access to school records.

Exclusion Criteria:

1. Parents or child unwilling to undergo the study surveys and assessments.

3.5 Study Definitions

3.5.1 Definition of Otoscopic findings

Otitis media for this study will be classified into discreet diagnoses based on physical examination findings and tympanometry. These categories are:

Normal - No evidence of ear disease

Mild Ear Disease

- **Acute otitis media (AOM)** – The presence of at least two of the following three signs: 1) white, yellow, amber, or blue tympanic membrane (TM; i.e., abnormal color), 2) opacification not due to scarring , 3) tympanometry peak compliance <0.2 ml or tympanometric width > 200 daPa on tympanometry; or bubbles or air-fluid interfaces; and one of the following three signs/symptoms: 1) ear pain, 2) redness of the TM, 3) fullness or bulging of the TM; or acute purulent otorrhea not due to otitis externa.
- **Otitis media with effusion (OME)** – The presence of at least two of the following three signs: 1) white, yellow, amber, or blue tympanic membrane (TM; i.e., abnormal color), 2) opacification not due to scarring , 3) tympanometry peak compliance <0.2 ml or tympanometric width > 200 daPa from tympanogram; or bubbles or air-fluid interfaces; and the absence of the following four signs/symptoms: : 1) ear pain, 2) redness of the TM, 3) fullness or bulging of the TM, 4) acute purulent otorrhea not due to otitis externa.
- **Healed Perforation –** Otherwise normal TM with a thinned out area of TM.
- **Myringosclerosis –** a hardening of the ear drum as calcium deposits form on the ear drum and middle ear; peak compliance on tympanogram > 5.0 ml.

Moderate Ear Disease

- **Dry Perforation –** perforation of the TM without otorrhea; or ear canal volume >=2 ml on tympanogram.
- **Adhesive otitis media –** Intact TM but retraction or retraction pocket seen.

Severe Ear Disease

- **Chronic Suppurative Otitis Media (CSOM), active –** perforation of the TM with otorrhea duration of more than 2 weeks; or TM with retraction pocket unable to see deepest part, with granulation tissue, squamous debris, or otorrhea.

3.5.2 Audiologic Evaluation

All children with abnormalities as listed above will be subjected to a formal **hearing test** at the Bohol Hearing Center (BHC). Children with potential hearing loss will be tested at the BHC and the level of hearing loss clearly documented.

Hearing Loss is defined as failure to hear the audiology tone at 15 decibels for 2 or more frequency levels. The frequency levels examined are 500, 1000, 2000, 4000, and 8000 Hz. In this study we include the 8000 Hz level, as language perception and acquisition is particularly impacted by impairment of the hearing at the higher frequencies.

Degree of hearing loss (WHO Criteria)[1] is defined as the average of four levels of hearing decibels at 500, 1000, 2000 and 4000 Hz in any ear as follows:

Degree of hearing loss	Decibels
Normal	<= 15
Mild	16 – 30
Moderate	31 to 60
Severe	61 to 80
Profound	>80

Types of hearing loss (HL) determination logic is illustrated in Figure 2. AC indicates the air conductance test and BC indicates the bone conductance test. “Mixed” HL is when there is evidence for both conductive and sensorineural HL.

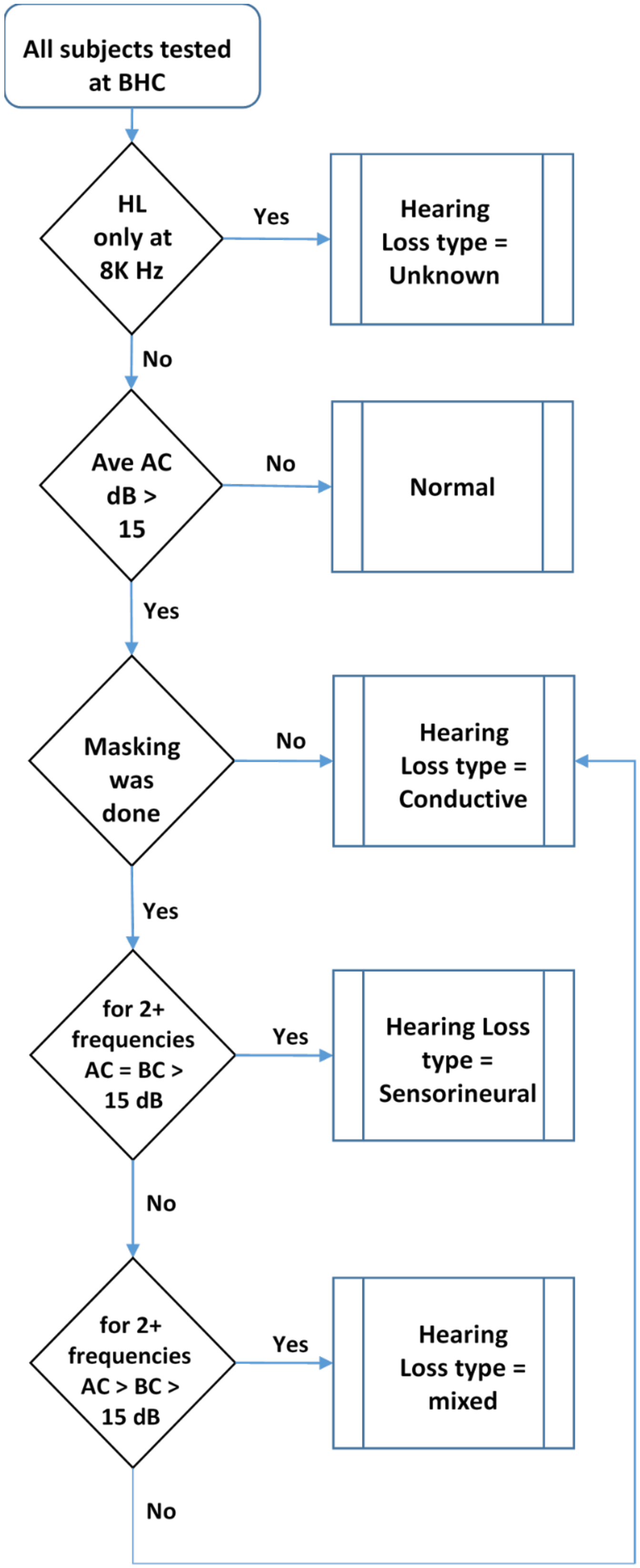

Disabling hearing loss refers to hearing loss greater than 40 decibels (dB) on average in the better ear. This is obtained by taking the average decibel level of the better ear at 500, 1000, 2000, and 4000 Hz per air conduction. In the event that there are hearing test results from the HearTest and Bohol Hearing Center (BHC), the BHC results will be used for analyses; if BHC results are not available the HearTest results will be used.

Hearing impairment is defined as disabling hearing loss (as defined above) and where BHC of HearTest were not done, failure in the HearScreen test.

Definitions of **types of hearing loss** (conductive, sensorineural or mixed) are shown in Figure 2.

Figure 2. Types of Hearing Loss

Data Quality Control/Quality Assurance Measures

4.1 Overall Study Data Quality Assurance- Personnel Roles

4.1.1. Study PI, Dr. Simões, visited the field site at least semiannually to ensure study progress and data quality. He was responsible for final study protocol, documents, IRB approvals and overall for data quality assurance

4.1.2. Philippine PIs Drs. Tallo and Lucero in conjunction with Dr. Simões were responsible for final study protocol, documents, IRB approvals and overall for data quality assurance oversight of study procedures in Philippines, to ensure that quality data results.

4.1.3. The Study Senior Research Associate/Study Coordinator, Diozele Sanvictores, has worked closely with the PIs and the US database manager/analyst to facilitate study communications, financial management, and data quality assurance.

4.1.4. The Study Senior Research Associate/Study Coordinator, Diozele Sanvictores, has met with the Filipino team and worked with the Filipino PIs, in conjunction with the Philippine database manager, to develop the study SOPs, to initiate the weekly (Bohol) study teleconference calls, and to monitor performance and quality of reporting.

4.1.5. The US database manager/analyst, Phyllis Carosone-Link, schedules biweekly team zoom teleconferences to discuss the ongoing research issues in data quality and facilitate study communications across the time zones.

4.1.6 Dr. Kenny Chan, Chairman of Children's Hospital Colorado ENT department, has trained the study nurses in the Philippines to do all of the ENT related protocols and procedures. In addition he supervises all of the ENT physician assistants in the US. He was responsible in conjunction with the study team, for developing the ENT study protocols. He reviews all of the abnormalities found in Philippines by the study nurses, when there is any question about whether subjects should be

referred to the ENT surgeon in Bohol or not. This data quality measure is described in a recent publication.[44]

4.1.7 Dr. Andrea Miele is a clinical psychologist. She went to the Philippines, and trained eight study nurses, in all of the methods required to do the psychometric testing, described in the section 3.3.5 above. This involved two weeks of on-site training including didactic lectures explaining the tests, then conducting the tests with the trainee nurses in the first week, followed by individual observation of trainees performing the tests on study subjects in the second week. Individual level assessment of the trainees was done.

4.2. Data Quality of Ear Disease Diagnoses

4.2.1. Dr. Kenny Chan, has trained 3 ENT Physician's Assistants (ENT PAs) to review the videos of the otoscopy that was performed in Philippines during the ear exam. Dr. Chan held two training sessions, and then an analysis was done to determine the agreement in detecting signs of ear disease (e.g., redness, scarring of the tympanic membrane, etc.) between the 3 ENT PAs and Dr. Chan. The analysis showed low sensitivity, and so a secondary training was done. During this evaluation process, the data collection form has been changed to clarify the signs that are to be noted. Some of the old descriptions of the signs were responsible for the low sensitivity scores. The 3 ENT PAs and Dr. Chan were given a second packet of videos to review after the 2nd training session, and the concordance will be analyzed using a Kappa score and a sensitivity analysis. Once the ratio of the weighted Cohen's **κ**: **κ max** reaches >.60, [46,47] the 3 ENT PAs will complete the review of the video otoscopy.

4.2.2. An algorithm has been developed to assign the diagnosis of ear diseases (e.g., CSOM, OME, etc.) in an objective manner. The algorithm brings together the signs that the ENTs

find from the video otoscopy review: the overt signs that are noted by the study nurses (e.g., otorrhea) and Philippine ENTs (e.g., otorrhea, tympanosclerosis, etc.), data from the subject's tympanogram and DPOAE results, and subject responses (i.e., ear pain, how long they have had otorrhea). The ear disease definitions in section 3.5.1 indicate these measures and how they will be applied to the diagnosis of the various types of ear disease. Using this algorithm improves the quality of the outcomes by ensuring that subjectivity is reduced.

4.3. Data Entry Quality Control

4.3.1. Abraham Sepulveda, the Philippine Access programmer, has developed the Filipino Access database to ensure data quality by:

4.3.1.1. Inserting range checks for numeric fields;

4.3.1.2. Programmed drop-down lists for allowable selections;

4.3.1.3. Made all numeric fields "required" so that only truly missing data are intentionally coded as such;

4.3.1.4. Establishing skip patterns to tie together contingent responses;

4.3.1.5. Linking all study data entry screens to a single subject ID to eliminate duplication potential;

4.3.2. Abraham Sepulveda and his team of Access programmers systematically check data quality on a monthly basis by running post validation programs in STATA (missing data and consistency checks). Errors listed are then emailed to the Study Senior Research Associate/Study Coordinator, Diozele Sanvictores for editing the Access database and re-uploading the edited database to REDCAP.

4.3.3. Thatcher Batista, the lead nurse for the ear exams, ensures that a Hear Test is performed for those subjects who are referred to an ENT and/or the Bohol Hearing Center

(BHC), as sometimes the subject does not return for their audiology screen and/or does not agree to be seen at the BHC. This way, we have some data on the worst cases from the ear exam, rather than none at all.

4.3.4. The results from the BHC audiology measures are quality checked by Phyllis Carosone-Link, who uploads these results into Redcap.

4.3.5. Phyllis Carosone-Link, the Redcap database administrator, has developed the Redcap (online) database, so that data from the Philippines Access database can be uploaded on a weekly basis. Redcap requirements and rules add an additional layer of data quality checks during the process of importing new data. For example, a mistyped subject ID number would be noted by Redcap before a data file import is confirmed.

4.3.6. The Study Senior Research Associate/Study Coordinator, Diozele Sanvictores monitors the weekly reports of what is uploaded by the Trackers into Redcap to ensure that all subjects included per barangay were covered by the Trackers in the field. For the school records entered in the computer and cognitive tests administered through the laptops, each record is checked for completeness and missingness. These practices ensure that the Access databases from the computers and in Redcap are identical.

4.3.7. The Redcap database administrator, systematically checks data quality on a quarterly basis in the Redcap database; this is done by downloading data into SAS to check for consistencies and any missing values. For example, those subjects who have had an ear exam are expected to also have data entered from the mechanical devises (tympanometer, HearScreen devise, etc.), unless there is a reason indicated that those measures cannot be taken (e.g., active ear disease with otorrhea).

4.3.8. The Redcap database administrator, has developed a Redcap "QC Log" in Redcap, where any of the investigators can upload needed data corrections. Each Redcap QC Log

entry, once corrected, remains “Unverified” until it is signed off as “Completed” by the person who uploaded the needed corrections. If corrections have not been completed to the reviewer’s satisfaction, the entry is marked as “Incomplete” and must go through the correction process again. Phyllis has created an SOP for this process.

4.3.9. Given the different nature of the cognitive outcome data, Dr. Andrea Miele has and will continue to download these data to ensure data quality. During the pre-testing phase, a cognitive battery was planned, translated, and administered to volunteers. Dr. Miele observed all administrations. Responses were scored and reviewed by Dr. Miele. Measures found to be unreliable or invalid were removed from the battery. During the initial months following study start, data quality control consisted of reviewing scoring responses with the Philippine test administrators. In addition, Dr. Miele used scanned raw data to verify items coded in the REDCap database. Dr. Miele also viewed videos of test administration by each tester and provided feedback. These videos are done randomly on all testers once a month to include complete examinations. These videos are reviewed by Dr Miele to assure that strict adherence to the established protocols for testing. She has been and remains available for scoring questions. More recently, Dr. Miele performed a descriptive data analysis for the study’s initial cognitive data, while rechecking data quality. She will be involved in the final data quality check on these data prior to the data analysis phase.

4.3.10. School performance (grades, absences, etc.) are double data-entered to ensure data accuracy for these measures. Discrepancies are flagged by the Philippine database manager on site and resolved by the two data entry operators.

Yubo Tan, working under the supervision of Elisabeth Root, the study statistician, using a turnkey SAS program, will ascertain 4.3.11. Semifinal and final data quality/consistency.

Statistical Methods

5.1 Sample size and power of study

Assuming a rate of OME plus CSOM of 3 to 5% in the unvaccinated children, we will need to follow-up and test between 3000 and 5000 of the former ARIVAC study participants. This will enable us to detect a 40% reduction in OME and CSOM with 80% power and a 2-tailed 5% false positive (alpha) rate. For 90% power, we would need between 5000 and 7000 study participants. We expect that the other study outcomes will be similarly powered to detect a vaccine effect, given the 10,000 subjects that we expect will participate in this study. Table 1 shows the current ages by district of the children who participated in the vaccine trial in 2000-2004, and districts where we conducted the GPS study in 2008.

For the mediated analysis, assuming a regression relationship of 0.25 between the mediator (the z-score of the WISC FSIQ) and the outcome (z-score of school performance in 6th grade) and a correlation of 0.5 between the predictor (CSOM/OME) and the mediator and a 2-tailed alpha of 0.05, we expect to have 86% power with a sample size of 5000 children. These estimates are based off an assumption of a ¼ of a standard deviation difference in school performance for each standard deviation increase in the WISC. For the mediated analysis of school dropout, assuming a regression coefficient of .1 between the mediator (z-score of WISC FSIQ) and the outcome (school dropout), a marginal prevalence of dropout of 20%, and a correlation of 0.5 between the predictor (CSOM/OME) and the mediator and a 2-tailed alpha of 0.05, we expect to have 80% power with a sample size of 5000 children.

5.2 Plan for handling missing data

Given the large amount of data collected as part of this project, there will undoubtedly be missing values in some items. We will handle missing data in the following ways:

a. Differences between children with missing and non-missing values will be examined across a range of child characteristics and outcomes to look for imbalances or biases.
b. For independent variables used in multivariate models, a "missing" category will be included as an explicit value in all categorized variables. For example, if there is a missing value when a parent is asked if the child has ever been diagnosed with asthma, the following categories will be used in analysis: Yes, No, Missing.
c. We will set a 5% threshold for missing values and if the number of children dropped from the analysis is below this threshold, we will assign a "missing" category as specified above. We will also drop these observations and examine the impact of reduced sample of effect sizes. If no significant change is detected we will continue with the proposed analysis.
d. As a robustness check, we will employ a model-based method for multiple imputation of missing independent variables using the multivariate imputation by chained equations method (MICE).[48,49] We will compare results across different methods of handling missing data to ensure that dropping observations and/or imputing data does not impact effect sizes.

5.3 Main trial endpoints

5.3.1 Demographic profile and evaluating balance between treatment groups

Baseline characteristics from the original PCV 11 trial database will be summarized for the children tracked into the current study. Maternal, child, and household characteristics from the current study database for the tracked cohort will be summarized using the parental questionnaire from the follow-up study.

5.3.2 Evaluating balance between vaccine groups

Although participants in our study were randomized at baseline, a variety of factors may have impacted the balance between the PCV 11 and placebo groups during the 10 years since the initial trial. This is a common problem with longitudinal studies – treatment groups often become less comparable over time due to loss to follow-up, missing data, and intervening health and economic conditions. In addition, since the primary purpose of the original trial was not to examine educational achievement, the treatment groups may not have originally been balanced on factors that directly impact this endpoint. To examine the balance between treatment groups we will examine factors derived from: a) demographic and health factors collected at baseline and b) demographic, health and SES factors collected on the parental questionnaire. Simple univariate methods such as t-tests, ANOVA and Chi-squared tests will be used to examine the difference in treatment groups. Non-parametric equivalents will be used when necessary. Simple univariate regression (logistic when appropriate) will also be used to examine simple differences in parent, household and child characteristics between the 11PCV and placebo groups. The alpha level for all tests will be set at 0.05. If any of these factors appear to affect the balance of the treatment groups with regard to the study endpoints, they will be included in multivariate models. Factors may include:

a. Demographic factors: birth order of child, household size, nuclear vs. extended family
b. Household socioeconomic status: maternal schooling and literacy, household consumption, income, presence of mother/father
c. Early-life stimulation: attendance in preschool, overall school attendance
d. Health: hearing loss, chronic health problems

5.3.3 Study endpoints

5.3.3.1. The primary analysis

Primary endpoints for the study are related to educational attainment. All math, science, English, and overall performance scores will be collected for each grade that the child attended. If a child fails one grade prior to moving to the next, the scores from the failing grade year will be used for analysis.

A. ***School Dropout***: whether or not a children dropped out of school (permanently) prior to completion of (6th grade)
B. ***Composite Scholastic Performance Scores***: all available scores for math, English, and science will be added up for two time periods: grades 1-3 and grades 1-6. Children who dropped out will receive a 0 for each year of schooling they should have completed after drop-out. Composite scores will then be converted into z-scores.
C. ***Mean Scholastic Performance Score:*** all available scores for math, English, and science will be added up for two time periods and divided by the number of years the child attended school: grades 1-3 and grades 1-6. Children who dropped out will receive a 0 for each year of schooling they should have completed after drop-out. Means will then be converted into z-scores.

5.3.3.2 Secondary analyses

We also specify a series of secondary outcomes which are considered subsidiary or exploratory in nature. These are broadly grouped into: additional educational outcomes, cognitive outcomes, and hearing and otoscopic outcomes.

A. ***Education Outcomes***

a) ***Composite Scholastic Subject Scores***: Composite scores for grades 1-3 and grades 1-6 will be calculated separately for:

   i) Mathematics Performance: all scores that are available for mathematics.

   ii) English Performance: all scores that are available for English.

   iii) Science Performance: all scores that are available for science.

b) ***Mean Scholastic Subject Score:*** Mean scores for grades 1-3 and grades 1-6 will be calculated separately for:

   i) Mathematics Performance

   ii) English Performance

   iii) Science Performance

c) ***Year of School Dropout***: indictor of year at which child dropped out

d) ***Individual Grade Composite Scholastic Performance Score:*** scores for all three subjects will be added up for each year. Scores will be converted into a grade normed z-score.

e) ***Individual Grade Mean Scholastic Performance Score:*** mean score of grades for all three subjects divided by the number of subjects the child had a score. Scores will be converted into a grade normed z-score.

f) ***Individual Grade Scholastic Performance Scores:*** the overall performance score given to a child for each subject at the end of each school year. Scores will be converted into a grade normed z-score for each year. Scores will be calculated separately for:

   i) Mathematics Scholastic Performance: Mathematics score available for each grade

   ii) English Scholastic Performance: English score available for each grade

   iii) Science Scholastic Performance: Science score available for each grade

g) ***Grade retention***: Number of years taken to complete 6th grade (excluding drop-outs).

h) ***Grade retention:*** Number of grades a child repeats for:

i) All years of schooling completed (max of 3 grades of repeat were recorded; we can examine whether a child repeated 1 grade, 2 grades, or 3 grades)

ii) During primary school

i) ***Attendance rate***: proportion of school days attended for primary and secondary schooling

j) ***Mean Overall Attendance Rate***: average yearly attendance rate (proportion of school days attended) across all years of schooling

B. Cognitive outcomes

A. **WISC-V Full scale IQ (FSIQ):** The full scale consists of 7 subtests that are summed to a full raw score, these are transformed into a z-score for analysis. Z-scores are constructed separately for 4 month age groups.

B. **WRAML Story Memory:** The number of details about the two stories a child lists are summed to create a raw score. There are two sets of questions collected for these stories: learning and memory. The raw score is transformed into a z-score which is constructed separately using 2 year age groups (e.g., 12 to <14, 14 to <16, 16 to <18, and 18 to <20). A z-score of two story tests (learning portion) and a z-score of two story tests (memory portion) are used.

C. **WRAML Verbal Learning:** The number of words a child lists are summed to a raw score. There are two sets of questions collected for these stories: learning and memory. Raw scores are transformed into z-scores which are constructed using 2 year age groups (e.g., 12 to <14, 14 to <16, 16 to <18, and 18 to <20). A z-score of the list test (learning portion) and a z-score of the list test (memory portion) are used.

D. CELF Following Directions: Responses for this subtest are summed to develop a raw score. The raw score is z-scores are constructed for 1 year age groups through age <17. Ages ≥ 17 can all be grouped for z-score calculation (through <21)

E. CELF Semantic Relationships: Responses to the subtest are summed to develop a raw score, which is transformed to a z-score. Z-scores are constructed for 1 year age groups through age <17. Ages ≥ 17 can all be grouped for z-score calculation (through <21)

F. BRIEF-2 GEC: Measures a child's day-to-day executive functioning skills. It is a summation of three indices created from the 8 subscales: inhibit, self-monitor, shift, emotional control, initiate, working memory, plan/organize, task-monitor, organization of materials. The raw summation is transformed into a z-score; z-scores are constructed for two age groups: 11 to <14 and 14 to <18.

G. CBCL Total Problems: The total problem score is a composite of 8 subscales which measure sets of behaviors that typically occur together. The raw score is transformed into a z-score which is constructed separately for child age 6 to <12 and 12 to <18.

H. WISC-V Verbal Comprehension Index (VCI): This index consists of 2 subtests summed to a raw score: similarities and vocabulary. Raw scores are transformed to z-scores separately by 4 month age groups.

I. WISC-V Visual Spatial Index (VSI): Responses for the one subtest for block designed are summed to a raw score. Raw scores are transformed to z-scores separately by 4 month age groups.

J. WISC-V Fluid Reasoning Index (FRI): The index consists of 2 subtests summed to a raw score: matrix reasoning and figure weights. Raw scores are transformed to z-scores separately by 4 month age groups.

K. **WISC-V Working Memory Index (WMI):** Responses for the one subtest for digit span are summed to a raw score. Raw scores are transformed to z-scores separately by 4 month age groups.

L. **WISC-V Processing Speed Index (PSI):** The scores from the one subtest on coding is are summed to a raw score. Raw scores are transformed to z-scores separately by 4 month age groups.

M. **WRAML Story Memory: story learning vs memory:** Difference between the two story memory subtests: learning vs. memory.

N. **WRAML Verbal Learning: verbal learning vs memory:** Difference between the two verbal learning subtests: learning vs. memory.

C. Hearing and Otoscopic outcomes

a) **Ear disease (specific)**: Chronic suppurative otitis media (active or inactive) or adhesive otitis media as defined by the signs and symptoms seen in the ear exam, the tympanometry and the video-otoscopy.

b) **Disabling hearing loss**: as defined by the BHC test or HearTest test results exclusive of profound hearing loss.

c) **Ear disease (broad)**: Mild, moderate or severe ear diseases as defined by the signs and symptoms seen in the ear exam, the tympanometry and the video-otoscopy.

d) **Hearing impairment**: defined as children with disabling hearing loss defined by the BHC test, or HearTest or who failed the HearScreen test.

e) **Hearing loss type**: defined as being conductive or mixed in etiology, specifically excluding sensorineural hearing loss.

5.3.4 Modeling methods

Table 3 displays the primary and secondary outcomes measured for this study and the statistical method used to examine the difference in treatment groups. Note that any children with cleft palette and CSOM will be excluded from the outcome analyses.

Table 3: Statistical Methods Proposed For Each Primary and Secondary Outcome	
**Outcome**	**Statistical Method**
**Primary Outcomes**	
School Dropout	Mediated hierarchical logistic regression
Composite Scholastic Performance	Mediated hierarchical linear regression
Mean Scholastic Performance	Mediated hierarchical linear regression
**Secondary outcomes**	
Overall composite and mean scholastic subject scores	Mediated hierarchical linear regression
Grade at dropout	Survival analysis
Yearly mean and composite scholastic performance, yearly subject scores	Mediated hierarchical longitudinal regression
Number of years taken to finish 6th grade	Mediated hierarchical regression
Number of repeated grades	Mediated hierarchical regression
WISC-V FSIQ, WISC-V subtests (VCI, VSI, FRI, WMI, PSI) WRAML story memory, WRAML verbal learning, CELF, BRIEF-2, CBCL	Mediated linear regression
Ear disease, disabling hearing loss, hearing impairment	Logistic regression

5.3.4.1 The primary analysis (school outcomes)

Simple univariate analyses will be conducted to compare the difference between PCV11 and placebo groups. Dropout rates will be calculated and chi-squared tests used to test the difference between groups. Means for composite and mean academic test score measures will be calculated and t-tests used to compare means. Since we are concerned about both mediation and treatment group balance, univariate analyses are not the primary statistical method of analysis and will be considered descriptive rather than confirmatory.

Primary study outcomes will be examined using mediated hierarchical regression modeling. Logistic models will be used for school dropout and linear models for scholastic performance measures. As our theoretical framework in Figure 1 shows, we anticipate the treatment effect on educational outcomes may be mediated by hearing, cognitive, and behavioral functioning. We also anticipate that children in

the same school may have correlated educational scores, and that other intervening factors over the 10 years since the initial trial may have both impacted the balance of the treatment groups and the outcomes themselves. To address all these concerns, we will use a mediated analysis. Multivariate models are necessary to examine confounding factors and treatment balance, which will be derived from the initial trial database and the current parental questionnaire. We categorize these external influences into:

a. Demographic factors: age, sex, birth order, maternal age at birth
b. Household socioeconomic status: maternal education, household consumption, income, household size, number of children in the household, presence of mother/father
c. Early-life stimulation: attendance in preschool, elementary school attendance
d. Health: asthma and other chronic health problems, 6 week height-for-weight z-scores, diagnosis of influenza, parainfluenza or RSV with pneumococcal colonization during ARIVAC trial

Second, we plan to use hierarchical regression models with random effects for school[50,51] to account for the clustering of children in schools and differentials between high-performing and low performing schools. We will also explore additional school effects gathered as part of the school database, including:

e. Type of school (e.g., public vs. private), attendance rate, grade retention, school-wide school performance z-scores.

Finally, we use mediation analysis to account for possible indirect or mediated effects of treatment on the outcome through both hearing, and cognitive/behavioral outcomes. Potential mediators are derived from the hearing and otoscopic tests and from the cognitive testing, including:

f. Health: hearing loss, ear disease,

g. Cognition: z-score for the WISC-V FSIQ, z-score for CELF and BRIEF, z-score for WRAML

h. Behavior: identified problem behaviors from the CBCL

We will conduct a mediation analysis using the product-of-coefficients method[52] to decompose the total effect of PCV11 vaccination into direct and indirect effects. The direct effect is the measurable effect of the PCV11 on the school outcome, while the indirect effect is the effect of the PCV11 when mediated by hearing and cognition. We will include an exposure-mediator interaction effect in each of the multivariate models and calculate the proportion-mediated measure which is the indirect effect divided by the total effect. Multiple mediators can be incorporated into these models and will be guided by our conceptual framework. We will conduct joint tests of significance of indirect and direct effects using the Sobel test or bootstrapping methods.[52,53]

For all univariate and multivariate analyses (in both the primary and secondary analyses), the alpha level will be set *a priori* at 0.05 with an entry level of 0.05. The recommended ratio of observations per variable of 10:1 will be adhered to. For linear models, R^2 will be presented to represent effect size and will be interpreted using standard descriptors.[54] For logistic models, the Hosmer-Lemeshow test and area under the ROC will be used for goodness of fit and discriminant analysis.[55] Models[56],[57],[58] will initially include vaccination status, then iteratively include blocks of variables that include confounding factors, school effects, and cognition/hearing mediators. If no mediation effects are detected the non-mediated model will be considered the final model.

5.3.4.2 Secondary analysis

A. Education Outcomes

As shown in Table 3, several of our secondary outcomes will also require the use of mediated multivariate hierarchical regression, which we will implement using the procedures outlined above. These include: overall composite and mean scholastic subject scores, number of years taken to finish 6th grade, number of repeated grades.

Other secondary education outcomes require a different modeling technique because of repeated measurement over time. These include: yearly mean and composite scholastic performance and yearly subject scores. For these outcomes, we will use trajectory-based modeling techniques. Three-level hierarchical longitudinal models[50,59] will be used for multi-year school performance variables to map the trajectory of school performance over time. In these models, multiple time points are nested within children who are nested within schools. These models examine the impact of treatment on initial (grade 1) school performance z-scores. They also model how school performance changes over time (slope of performance from 1st to 6th grade).[51] Random effects for school and other school effects will be included to control for potential clustering of school performance among children attending the same school. The strength of these models is that they do not require data points for every year for each child. They also allow for changing school effects as children move between schools. Longitudinal growth curve models also allow for mediated effects, so will include cognitive, behavioral, and hearing effects. If no mediation exists, we will consider the non-mediated growth models the final models.

The secondary outcome for year of school dropout will necessitate a survival analysis, as this is a "time to event" outcome variable. We will estimate simple Kaplan-Meier survival curves for the two treatment groups and compare them using the log-rank test. We will use Cox proportional hazards

regression models to examine the probability that children drop out prior to completion of 6th grade, and whether there is a difference in this probability between treatment groups. We will assess whether the proportional hazards assumption is reasonable using standard methods.[60] Using multivariate survival models allow us to examine what factors may modify the survival probability and can also incorporate effect modification through interaction terms.

B. Cognitive outcomes

All cognitive outcomes are calculated as z-scores. Univariate analyses (t-tests) will be used to compare the mean z-scores of each of the cognitive measures between the PCV11 and placebo groups. Given the nature of cognitive functioning, additional simple statistical analyses will examine the contribution of specific domains to the overall cognitive measure in addition to vaccination. The specific domains we will examine for each measure are outlined below:

1. WISC-V FSIQ
    a. Difference accounted for by VCI, WMI, BRIEF GEC, and CBCL Total Problems
2. WRAML Story Memory
    a. Difference accounted for by WISC-V WMI, WRAML Sentence Memory, CELF Following Directions, WRAML Picture Memory
3. WRAML Verbal Learning
    a. Difference accounted for by WMI, WRAML Sentence Memory, CELF Following Directions, WRAML Picture Memory
4. CELF Following Directions
    a. Difference accounted for by WISC-V WMI
5. CELF Semantic Relationships
    a. Difference accounted for by WISC-V VCI

6. BRIEF-2 GEC
    a. Difference accounted for by BRI, ERI, and CRI
7. CBCL Total Problems
    a. Difference accounted for by Internalizing and Externalizing problems

Mediated multivariate regression analysis will be used to predict each cognitive endpoint. Executive functioning and behavioral domains may be potential confounders or may mediate the impact of treatment on cognition. The following domains will be examined for confounding and mediation.

- CELF following direction
- CELF sematic relationships
- BRIEF-2 GEC – also subscales BRI, ERI and CRI
- CBCL total problems – also internalizing and externalizing subscales

Mediation analysis of cognitive outcomes will be conducted using the same procedure outlined in Section 5.3.4.1 above.

C. **Hearing and Otoscopic Outcomes**

Simple univariate analyses will be conducted to compare the difference between PCV11 and placebo groups in hearing and otoscopic outcomes. Rates of ear disease, disabling hearing loss, and degree and type of hearing loss will be calculated and chi-squared tests used to test the difference between groups.

Multivariate logistic regression analysis will be used to compare hearing impairment, ear disease and hearing loss between PCV11 and placebo groups while controlling for potential confounders. Vaccine efficacy will be computed with 95% confidence intervals.

5.4 Subgroup/Stratified Analysis

We will conduct a subgroup analyses to look for nonrandom variability in the direction or magnitude of a treatment effect. Previous research on this population at baseline indicated that both socioeconomic status (as measured by maternal education) and distance to health services modified the effect of the PCV11 treatment. Therefore, we will conduct a test for interaction to evaluate if these subgroup variables have a statistically significant interaction with the treatment indicator.[61] If the interaction is significant, then the treatment effect will be estimated separately at each level of the categorical variable used to define mutually exclusive subgroups. We explicitly define the following subgroups:

A. **Socioeconomic class**: Using the consumption data from the household questionnaire, we will construct a measure of household wealth. This measure we be categorized (e.g., quintiles) and children in each category compared.
B. **Urban/rural**: Using the GPS data collected during the household survey, each child will be classified by urban/peri-urban/rural/isolated rural and children in each category compared.
C. **Access to Bohol Regional Hospital (BRH):** Using the GPS data, each child we be assigned a distance from their household to in Tagbilaran. This measure will be categories (e.g., quintiles) and children in each category compared. We expect the treatment effect to be greater for children living further from BRH.

References

1. Deafness and Hearing Loss. WHO, 2018. (Accessed 28 November, 2018, at http://www.who.int/news-room/fact-sheets/detail/deafness-and-hearing-loss.)
2. Acuin J. Chronic suppurative otitis media : burden of illness and management options. Geneva: World Health Organization; 2004.
3. Schilder AG, Chonmaitree T, Cripps AW, et al. Otitis media. Nature reviews Disease primers 2016;2:16063.
4. Gell FM, White EM, Newell K, et al. Practical screening priorities for hearing impairment among children in developing countries. Bull World Health Organ 1992;70:645-55.
5. Jung TT, Alper CM, Hellstrom SO, et al. Panel 8: Complications and sequelae. Otolaryngol Head Neck Surg 2013;148:E122-43.
6. Margolis RH, Saly GL, Hunter LL. High-frequency hearing loss and wideband middle ear impedance in children with otitis media histories. Ear Hear 2000;21:206-11.
7. Roland PS. Chronic suppurative otitis media: a clinical overview. Ear Nose Throat J 2002;81:8-10.
8. Prymula R, Bergsaker MR, Esposito S, et al. Protection against varicella with two doses of combined measles-mumps-rubella-varicella vaccine versus one dose of monovalent varicella vaccine: a multicentre, observer-blind, randomised, controlled trial. Lancet 2014;383:1313-24.
9. Schuerman L, Borys D, Hoet B, Forsgren A, Prymula R. Prevention of otitis media: now a reality? Vaccine 2009;27:5748-54.
10. Leach AJ, Wigger C, Hare K, et al. Reduced middle ear infection with non-typeable Haemophilus influenzae, but not Streptococcus pneumoniae, after transition to 10-valent pneumococcal non-typeable H. influenzae protein D conjugate vaccine. BMC Pediatr 2015;15:162.
11. Sartori AL, Minamisava R, Bierrenbach AL, et al. Reduction in all-cause otitis media-related outpatient visits in children after PCV10 introduction in Brazil. PLoS One 2017;12:e0179222.
12. Sigurdsson S, Eythorsson E, Hrafnkelsson B, Erlendsdottir H, Kristinsson KG, Haraldsson A. Reduction in All-Cause Acute Otitis Media in Children <3 Years of Age in Primary Care Following Vaccination With 10-Valent Pneumococcal Haemophilus influenzae Protein-D Conjugate Vaccine: A Whole-Population Study. Clin Infect Dis 2018;67:1213-9.
13. Dagan R, Pelton S, Bakaletz L, Cohen R. Prevention of early episodes of otitis media by pneumococcal vaccines might reduce progression to complex disease. Lancet Infect Dis 2016;16:480-92.
14. Wiig EH, Semel E, Secord WA. Clinical Evaluation of Language Fundamentals. Fifth ed. Bloomington MN: PolyCorp; 2013.
15. Puleston R, Bugg G, Hoschler K, et al. Multi-centre observational study of transplacental transmission of influenza antibodies following vaccination with AS03(A)-adjuvanted H1N1 2009 vaccine. PLoS One 2013;8:e47448.
16. Cunningham AE, Stanovich KE. Early reading acquisition and its relation to reading experience and ability 10 years later. Dev Psychol 1997;33:934-45.
17. Kindig JS, Richards HC. Otitis media: precursor of delayed reading. J Pediatr Psychol 2000;25:15-8.
18. Bess FH, Dodd-Murphy J, Parker RA. Children with minimal sensorineural hearing loss: prevalence, educational performance, and functional status. Ear Hear 1998;19:339-54.
19. Culbertson JL, Gilbert LE. Children with unilateral sensorineural hearing loss: cognitive, academic, and social development. Ear Hear 1986;7:38-42.

20. Marschark M, Shaver DM, Nagle KM, Newman LA. Predicting the Academic Achievement of Deaf and Hard-of-Hearing Students From Individual, Household, Communication, and Educational Factors. Except Child 2015;81:350-69.
21. Jarvelin MR, Maki-Torkko E, Sorri MJ, Rantakallio PT. Effect of hearing impairment on educational outcomes and employment up to the age of 25 years in northern Finland. Br J Audiol 1997;31:165-75.
22. Fabelo T, Austin J, Gunter A. The Impact of Ignoring Dyslexia and Reading Disabilities in the Criminal Justice System: What We Know and Need to Know. Washington DC: JFA Associates; 2004:1-91.
23. Roberts JE, Burchinal MR, Campbell F. Otitis media in early childhood and patterns of intellectual development and later academic performance. J Pediatr Psychol 1994;19:347-67.
24. Roberts JE, Burchinal MR, Zeisel SA, et al. Otitis media, the caregiving environment, and language and cognitive outcomes at 2 years. Pediatrics 1998;102:346-54.
25. Roberts JE, Burchinal MR, Collier AM, Ramey CT, Koch MA, Henderson FW. Otitis media in early childhood and cognitive, academic, and classroom performance of the school-aged child. Pediatrics 1989;83:477-85.
26. Bennett KE, Haggard MP. Behaviour and cognitive outcomes from middle ear disease. Arch Dis Child 1999;80:28-35.
27. Bennett KE, Haggard MP, Silva PA, Stewart IA. Behaviour and developmental effects of otitis media with effusion into the teens. Arch Dis Child 2001;85:91-5.
28. Teele DW, Klein JO, Chase C, Menyuk P, Rosner BA. Otitis media in infancy and intellectual ability, school achievement, speech, and language at age 7 years. Greater Boston Otitis Media Study Group. J Infect Dis 1990;162:685-94.
29. Paradise JL, Dollaghan CA, Campbell TF, et al. Otitis media and tympanostomy tube insertion during the first three years of life: developmental outcomes at the age of four years. Pediatrics 2003;112:265-77.
30. Paradise JL, Dollaghan CA, Campbell TF, et al. Language, speech sound production, and cognition in three-year-old children in relation to otitis media in their first three years of life. Pediatrics 2000;105:1119-30.
31. Hall AJ, Maw R, Midgley E, Golding J, Steer C. Glue ear, hearing loss and IQ: an association moderated by the child's home environment. PLoS One 2014;9:e87021.
32. Mortensen M, Nielsen RB, Fisker N, Norgaard M. Hospitalisation with otitis media in early childhood and cognitive function in young adult life: a prevalence study among Danish conscripts. BMC Pediatr 2013;13:8.
33. Mody M, Schwartz RG, Gravel JS, Ruben RJ. Speech perception and verbal memory in children with and without histories of otitis media. J Speech Lang Hear Res 1999;42:1069-79.
34. Nittrouer S, Burton LT. The role of early language experience in the development of speech perception and phonological processing abilities: evidence from 5-year-olds with histories of otitis media with effusion and low socioeconomic status. J Commun Disord 2005;38:29-63.
35. Dewey D, Wall K. Praxis and memory deficits in language-impaired children. Dev Neuropsychol 1997;13:507-12.
36. Sheslow D, Adams W. Wide Range Assessment of Memory and Learning, Second Edition administration and technical manual. Wilmington, DE: Wide Range; 2003.
37. Achenbach TM. The Achenbach System of Empirically Based Assessemnt (ASEBA): Development, Findings, Theory, and Applications. Burlington VT: University of Vermont Research Center for Children, Youth, & Families.; 2009.
38. Gioia GA, Isquith PK, Guy SC, Kenworthy L. Behavior rating inventory of executive function. Child Neuropsychol 2000;6:235-8.

39. Lucero MG, Nohynek H, Williams G, et al. Efficacy of an 11-valent pneumococcal conjugate vaccine against radiologically confirmed pneumonia among children less than 2 years of age in the Philippines: a randomized, double-blind, placebo-controlled trial. Pediatr Infect Dis J 2009;28:455-62.
40. Wechlser D. The Wechsler Intelligence Scale for Children. Bloomington, MN: PolyCorp; 2014.
41. Tanskanen A, Nillos LT, Lehtinen A, et al. Geographic Information System and tools of spatial analysis in a pneumococcal vaccine trial. BMC Res Notes 2012;5:51.
42. Root ED, Lucero M, Nohynek H, et al. Distance to health services affects local-level vaccine efficacy for pneumococcal conjugate vaccine (PCV) among rural Filipino children. Proc Natl Acad Sci U S A 2014;111:3520-5.
43. Thomas DS, Anthamatten P, Root ED, et al. Disease mapping for informing targeted health interventions: childhood pneumonia in Bohol, Philippines. Trop Med Int Health 2015.
44. Chan KH, Dreith S, Uhler KM, et al. Large-scale otoscopic and audiometric population assessment: A pilot study. Int J Pediatr Otorhinolaryngol 2018;117:148-52.
45. Harris PA, Taylor R, Thielke R, Payne J, Gonzalez N, Conde JG. Research electronic data capture (REDCap)--a metadata-driven methodology and workflow process for providing translational research informatics support. Journal of biomedical informatics 2009;42:377-81.
46. Brenner H, Kliebsch U. Dependence of weighted kappa coefficients on the number of categories. Epidemiology 1996;7:199-202.
47. Sim J, Wright CC. The kappa statistic in reliability studies: use, interpretation, and sample size requirements. Phys Ther 2005;85:257-68.
48. Rubin D. Multiple Imputation after 18+ Years (with Discussion). Journal of the American Statistical Association 1996;91:473–89.
49. van Buuren S, Oudshoorn K. Flexible mutlivariate imputation by MICE. Leiden: TNO; 1999 1999-01-01.
50. Bryk AS, Raudenbush SW. Application of hierarchical linear models to assessing change. Psychological bulletin 1987;101:147.
51. Rumberger RW, Thomas SL. The distribution of dropout and turnover rates among urban and suburban high schools. Sociology of education. Sociology of Education 2000;73:39-67.
52. Hayes AF. Introduction to mediation, moderation, and conditional process analysis: A regression-based approach, 2nd Edition. New York, NY: Guilford Publications; 2018.
53. Preacher KJ, Hayes AF. Asymptotic and resampling strategies for assessing and comparing indirect effects in multiple mediator models. Behav Res Methods 2008;40:879-91.
54. Cohen J. Statistical power analysis for the behavioral sciences (2nd ed.). . Hillsdale, NJ: Lawrence Erlbaum Associates; 1988.
55. Hosmer DW, Lemeshow S. (2013). Applied Logistic Regression, 3rd Ed. New York: John Wiley and Sons; 2013.
56. Raudenbush SW, Bryk AS. Hierarchical linear models: Applications and data analysis methods. : Sage Publications Inc.; 2002.
57. Webster WJ, Mendro RL, Orsak TH. An Application of Hierarchical Linear Modeling to the Estimation of School and Teacher Effect. Wash, D.C.: ERIC Clearinghouse; 1998.
58. Gelman A, Hill J. Data analysis using regression and multilevel/hierarchical models. Cambridge, MA: Cambridge university press; 2006.
59. Singer JD, Willett JB. Applied Longitudinal Data Analysis: modeling change and event occurrence. New York, NY: Oxford University Press; 2003.
60. Hosmer DW, Lemeshow S. Applied Survival Analysis: Regression Modeling of Time to Event Data. NewYork, NY: John Wiley and Sons; 1999.

61. Varadhan R, Seeger JD. Estimation and Reporting of Heterogeneity of Treatment Effects. Developing a Protocol for Observational Comparative Effectiveness Research: A User's Guide. Rockville, MD: Agency for Healthcare Research and Quality; 2013.

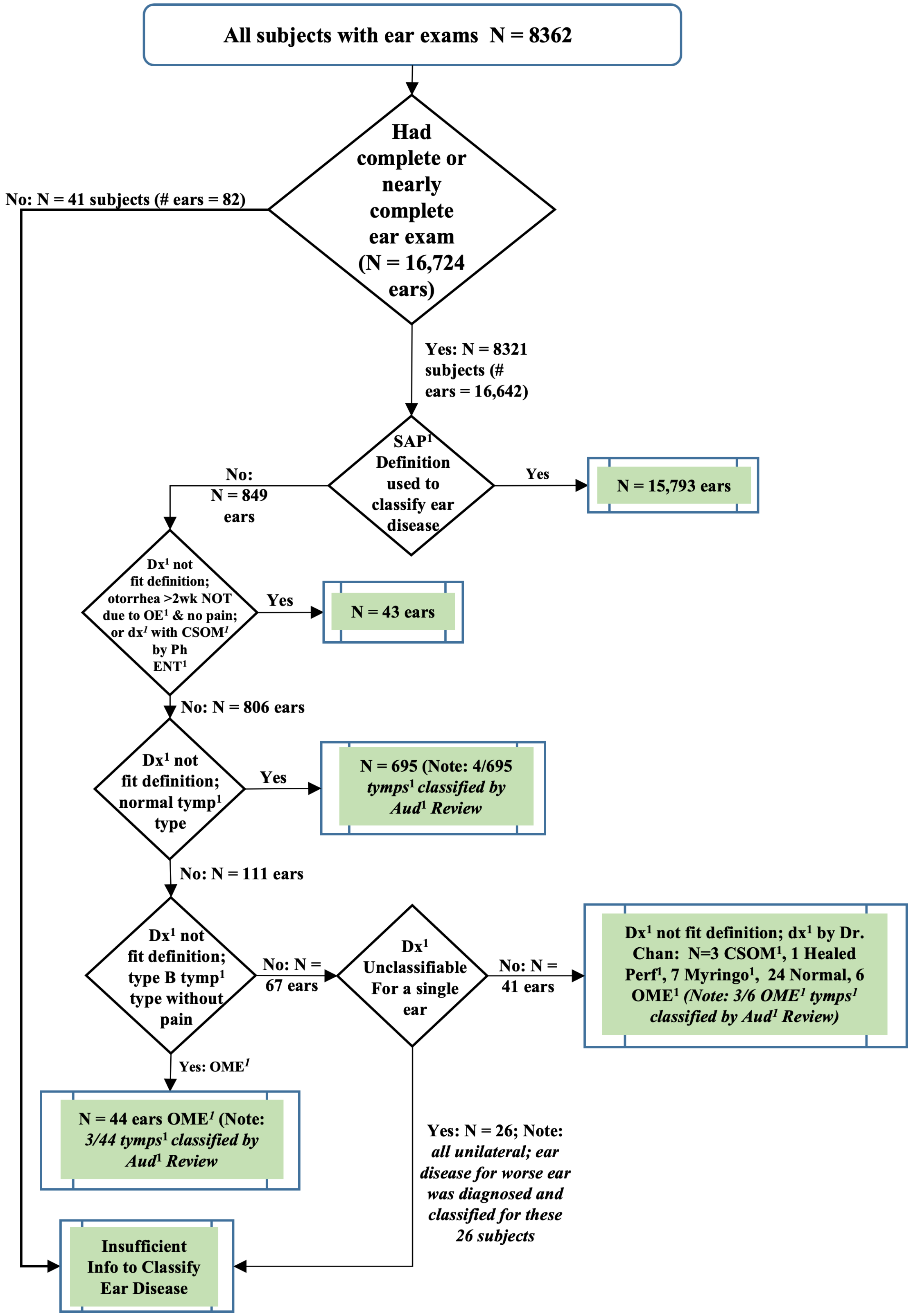

[1]Abbrieviations:
SAP = Statistical Analysis Plan; Dx = Diagnosed/Diagnosis; OE = Otitis Externa; tymp = tympanogram; Ph ENT = Philippine ENT doctor; CSOM = Chronic Suppurative Otitis Media; OME = Otitis Media with Effusion; Aud Review = Audiology Review; Perf = Perforation; Myringo = Myringosclerosis

Appendix Figure 1: Subjects with Complete or Partial Ear Exams and Methodology for Classification of Ear Disease

Appendix Methods

Statistical Analyses: Comparisons of demographic characteristics were made by follow-up status groups, to determine whether the current 11PCV study population was representative of the original ARIVAC RCT study population. Comparisons were also made between 11PCV and of study participants. Finally, comparisons were made between the 11PCV and placebo groups for the primary outcomes of this analysis, ear disease and hearing loss severity, and subgroups of the combination of ear disease and hearing loss.

Comparisons by follow-up status: Continuity-Adjusted Chi-squared tests were used to compare categorical demographic characteristics between children who were followed-up for the current 11PCV study and those who were not found. Student's *t*-tests were used to compare follow-up status groups for continuous variables. Median tests were also used, to validate the *t*-test results.

Comparisons by vaccine status: Demographic attributes and potential risk factors derived from the subject Case Report Form were analyzed by vaccine/placebo groups. Continuous variables were analyzed using a Student's *t*-test. The socioeconomic index used was computed using the Demographic and Health Surveys (DHS) Wealth Index. Continuity-Adjusted Chi-squared tests were used to compare categorical variables, with the exception of when a Fisher's Exact test was needed. A *P*-value < 0·05 was used as the significance threshold.

Comparisons between the 11PCV and placebo groups for the study outcomes. Relative and absolute Risk Reductions (RRR and ARR) with 95% confidence intervals (CIs)[20] with Yates' Continuity adjustment for outcomes in the 11 PCV group relative to the control group were computed using Javastat (https://statpages.info/ctab2x2.html). The primary outcome consisted of measurement of the reduction in moderate to severe ear disease in children who had previously received the 11 PCV compared to those who had not. The secondary hearing outcomes compared mild or moderate to severe hearing loss in the worst ear between the 11 PCV group and the controls. In children with moderate to severe ear disease the comparison of the relative reductions in mild or moderate/severe hearing loss between the 2 groups comprised the tertiary outcome.

Association between OM diagnosed in the ARIVAC study and ear disease classification in the current 11PCV study. Diagnostic codes for OM (CSOM, OME, and AOM) were included in the ARIVAC database for those children who were hospitalized or were seen in a study clinic. These OM results were compared to the OM results in the current 11PCV study, for those children who participated in the current study, using odds ratios and their 95% CI.

Comparing Hearing Loss in the Worst Ear by Type of Ear Disease. Descriptive statistics (mean, standard deviation, median, inter-quartile range) of PTA levels for children with and without hearing loss were compared using Wilcoxon Rank Sum tests, separately, by type of moderate to severe ear disease.

Attribute During ARIVAC Study		Total N	11PCV Study Follow-up: Child Found (N = 8926), N (%)	Child Not Found (N = 3099), but presumed alive N (%)	*P*-Value[1]
Child's Year of birth:	2000	2507	1790 (20.0)	717 (23.1)	***0.0003***
	2001	3575	2641 (29.6)	934 (30.1)	0.58
	2002	3429	2556 (28.6)	873 (28.2)	0.64
	2003	2514	1939 (21.7)	575 (18.6)	***0.0002***
Urban Residence during ARIVAC		5723	3996 (44.8)	1727 (55.7)	***< 0.0001***
Rural Residence during ARIVAC		6302	4930 (55.2)	1372 (44.3)	
Male Sex[2]		6248	4610 (51.7)	1638 (52.9)	0.25
Female Sex[2]		5777	4316 (48.4)	1461 (47.1)	
Residence during ARIVAC:					
	Tagbilaran	5723	3996 (44.8)	1727 (55.7)	***< 0.0001***
	Dauis	2175	1725 (19.3)	450 (14.5)	***< 0.0001***
	Panglao	1409	1140 (12.8)	269 (8.7)	***< 0.0001***
	Baclayon	817	615 (6.9)	202 (6.5)	0.50
	Cortes	868	652 (7.3)	216 (7.0)	0.56
	Balilihan	1033	798 (8.9)	235 (7.6)	***0.0202***
Mother employed		2667	1902 (21.3)	765 (24.7)	***0.0001***
Mother unemployed		9358	7024 (78.7)	2334 (75.3)	

Appendix Table 1: Demographic and Medical Characteristics by 11PCV Study Follow-Up Status

[1] Continuity-Adjusted Chi-Squared; [2] Male/Female designation was given by the parents of the infants during enrollment for the ARIVAC study. Sex was verified during enrollment for the current study.

	11PCV[1] Study Follow-up Status							
	Child Found			Child Not Found				
**Family Attribute**	**N**	**Mean (SD[2])**	**Median (IQR[3])**	**N**	**Mean (SD[2])**	**Median (IQR[3])**	***P*-Value[4]**	**Median Test *P*-Value**
Number of live siblings at enrollment	8926	0.83 (0.83)	1.0 (0, 1.0)	3099	0.72 (0.79)	1.0 (0, 1.0)	<0.0001	<0.0001
Mother's Education (# grades + # years vocational school + # year higher education)	8926	10.2 (3,5)	10.0 (7.0, 13)	3099	10.9 (3.6)	10.0 (8.0, 15)	<0.0001	<0.0001

Appendix Table 2: Descriptive Analysis of Demographics Characteristics by 11PCV[1] Study Follow-up Status (Continuous Variables)

[1]11PCV: 11-Valent Pneumococcal Vaccine; [2] SD = standard deviation; [3] IQR = Interquartile range; [4] Student's *t*-Test results

	11PCV[1] N/vaccine N (%)	Placebo N/placebo N (%)	*P*-value[2]
Ethnicity			
Cebuano/Boholano/Tagalog	81/172 (47.1)	91/172 (52.9)	
Cebuano/Boholano/Ilocano	77/142 (54.2)	65/142 (45.8)	
Cebuano/Boholano/Hiligaynon Ilonggo	33/67 (49.3)	34/67 (50.8)	
Cebuano/Boholano/Bikol	25/48 (52.1)	23/48 (47.9)	
Cebuano/Boholano/Waray	22/42 (52.4)	20/42 (47.6)	
Cebuano/Boholano	3116/6206 (50.2)	3090/6206 (49.8)	0.35
Cebuano/Boholano/Visayan	608/1218 (49.9)	610/1218 (50.1)	
Visayan	56/111 (50.5)	55/111 (49.6)	
Moslem	13/19 (68.4)	6/19 (31.6)	
Badjao	13/23 (56.5)	10/23 (43.5)	
Other	1/10 (10.0)	9/10 (90.0)	

Appendix Table 3: Descriptive Analysis of Ethnicity by Vaccine Status

[1]11-Valent Pneumococcal Vaccine; [2]Pearson Chi-squared *P*-value

Vaccine Status	Normal	Mild	Moderate	Severe	Total	*P*-Value[1]
11PCV[2]	2379 (56.8%)	1499 (35.8%)	255 (6.1%)	55 (1.3%)	4188	0.28
Placebo	2282 (55.2%)	1495 (36.2%)	290 (7.0%)	66 (1.6%)	4133	

Appendix Table 4: Ear Disease Severity in Worst Ear N (%)

[1]Pearson's Chi-square *P*-value;
[2]11-Valent Pneumococcal Vaccine

I

Vaccine Status	Normal to Mild Ear Disease[1]	Components of Moderate to Severe Ear Disease				
		Dry Perforation	Adhesive OM[2]	CSOM[3]	Total	*P*-Value[4]
11PCV[5]	3878 (92.6%)	65 (1.6%)	190 (4.5%)	55 (1.3%)	4188	0.23
Placebo	3777 (91.4%)	71 (1.7%)	219 (5.3%)	66 (1.6%)	4133	

Appendix Table 5: Ear Disease in Worst Ear by Vaccine Status: Components of Moderate and Severe Ear Disease

[1]No Ear Disease (Normal), Acute Otitis Media, Otitis Media with Effusion, Healed Perforation, or Myringosclerosis; [2]Otitis Media; [3]Chronic Suppurative Otitis Media; Pearson's Chi-square *P*-value; [5]11-Valent Pneumococcal Vaccine

Vaccine Status	Hearing Loss Severity in the Worst Ear N (%) Normal (0 – 15 dB)	Mild (16 – 30 dB)	Moderate (31 – 60 dB)	Severe (61 – 80 dB)	Profound (>80 dB)	Total	*P*-Value[1]
**11PCV[2]**	3802 (92.0)	225 (5.5)	89 (2.2)	7 (0.2)	8 (0.2)	4131	0.15
**Placebo**	3746 (91.8)	246 (6)	67 (1.6)	7 (0.2)	16 (0.4)	4082	

Appendix Table 6: Hearing Loss Severity by Vaccine Status

[1]Pearson's Chi-square *P* -value; [2]11-Valent Pneumococcal Vaccine
